# Enhancing learning experiences: EEG-based passive BCI system adapts learning speed to cognitive load in real-time, with motivation as catalyst

**DOI:** 10.3389/fnhum.2024.1416683

**Published:** 2024-10-07

**Authors:** Noémie Beauchemin, Patrick Charland, Alexander Karran, Jared Boasen, Bella Tadson, Sylvain Sénécal, Pierre-Majorique Léger

**Affiliations:** ^1^Tech3Lab, HEC Montréal, Information Technology Department, Montreal, QC, Canada; ^2^Didactics Department, Université du Québec à Montréal, Montreal, QC, Canada; ^3^Faculty of Health Sciences, Hokkaido University, Sapporo, Japan

**Keywords:** brain-computer interface, passive BCI, adaptive interface, EEG, neuroadaptive, learning, computer-based learning, cognitive load

## Abstract

Computer-based learning has gained popularity in recent years, providing learners greater flexibility and freedom. However, these learning environments do not consider the learner’s mental state in real-time, resulting in less optimized learning experiences. This research aimed to explore the effect on the learning experience of a novel EEG-based Brain-Computer Interface (BCI) that adjusts the speed of information presentation in real-time during a learning task according to the learner’s cognitive load. We also explored how motivation moderated these effects. In accordance with three experimental groups (non-adaptive, adaptive, and adaptive with motivation), participants performed a calibration task (*n*-back), followed by a memory-based learning task concerning astrological constellations. Learning gains were assessed based on performance on the learning task. Self-perceived mental workload, cognitive absorption and satisfaction were assessed using a post-test questionnaire. Between-group analyses using Mann–Whitney tests suggested that combining BCI and motivational factors led to more significant learning gains and an improved learning experience. No significant difference existed between the BCI without motivational factor and regular non-adaptive interface for overall learning gains, self-perceived mental workload, and cognitive absorption. However, participants who undertook the experiment with an imposed learning pace reported higher overall satisfaction with their learning experience and a higher level of temporal stress. Our findings suggest BCI’s potential applicability and feasibility in improving memorization-based learning experiences. Further work should seek to optimize the BCI adaptive index and explore generalizability to other learning contexts.

## 1 Introduction

Computer-Based Learning (CBL) is an educational approach that uses computer software to deliver, assist, and enhance the learning processes (Grizioti and Kynigos, 2020). The CBL environment learners use in their learning can take multiple forms, such as programs, applications, tools, and platforms (Grizioti and Kynigos, 2020). CBL provides students with instant feedback, individualized learning paths and greater flexibility, all of which can increase student engagement and comprehension (Grizioti and Kynigos, 2020; Mertens et al., 2022; Van der Kleij et al., 2015). As a result, CBL is increasingly used in educational programs as an important complement to conventional classroom teaching or as a stand-alone pedagogical method (Grizioti and Kynigos, 2020).

However, offering access to CBL does not guarantee a successful learning experience. For example, online courses allow many more students to enroll because the number of physical seats available in the classroom does not limit their capacity. Moreover, their accessibility makes it possible to take the course at any time, from anywhere in the world. Because of their greater capacity and the diversity of students enrolled in these online courses, the vast majority of online courses have been developed using the classic “one size fits all” approach, with little to no consideration of individual differences and learning abilities (Tekin et al., 2015; Wang and Lehman, 2021). In addition, the distance between the teacher and the students in CBL makes the assessment of learning needs and abilities even more difficult (Tekin et al., 2015). As a result, this can lead to low levels of learning engagement (Bawa, 2016; Dumford and Miller, 2018) and motivation (Ferrer et al., 2022; Fini, 2009; Mamolo, 2022; Wang and Lehman, 2021) among learners.

The need to tailor the learning experience to the individual learner has been observed, mentioned, and studied many times in the current literature (Klašnja-Milićević et al., 2011; Mutlu-Bayraktar et al., 2019; Tekin et al., 2015; Wu et al., 2020). In educational psychology, the concept of the Zone of Proximal Development (ZPD) developed by Lev Vygotsky draws the theoretical foundations that support personalized learning (Chaiklin, 2003; Tetzlaff et al., 2021; Vygotsky and Cole, 1978). This concept emphasizes the need to understand that each learner is at a different point in their cognitive development. According to Vygotsky, the ZPD represents the set of tasks or skills that a learner cannot yet perform alone but can perform with assistance (Vygotsky and Cole, 1978). Learning is not encouraged by tasks that are too simple or already within the scope of our current abilities, leading to a state of boredom (Vygotsky and Cole, 1978). Conversely, no learning occurs when tasks are overly complex and frustrating tasks that exceed our abilities (Vygotsky and Cole, 1978). Thus, maintaining a learner’s ZPD provides the ideal level of challenge to promote growth and development, which can be further enhanced by personalized support and guidance to improve academic performance over traditional “one-size-fits-all” teaching methods (Alamri et al., 2021).

Complementary to the ZPD, the concept of cognitive load is important for understanding and personalizing learning experiences (Mutlu-Bayraktar et al., 2019; Sweller, 2020; van Merriënboer and Ayres, 2005). Cognitive Load Theory (CLT) examines human cognitive architecture and provides insight into how learners process and retain information in memory (Curum and Khedo, 2021; Sweller, 1988; Sweller et al., 1998; Wouters et al., 2008). This theory considers the interplay between the working memory’s limited capacity and long-term memory (Kalyuga and Liu, 2015; Mutlu-Bayraktar et al., 2019). It defines cognitive load as the mental workload required to perform a learning task and emphasizes the importance of managing the mental effort required for effective learning (Kalyuga and Liu, 2015; Zhou et al., 2017a). Thus, performing a learning task requiring too much or too little mental effort will lead to less-than-optimal learning experiences and poor performances (De Jong, 2010). In a CBL environment, ZPD can serve as a tool to tailor educational tasks and support to suit the learner’s abilities, helping maintain cognitive load at an optimal level while learning. Unfortunately, current CBL environments only consider the learner’s perceived cognitive load as a global design consideration, disregarding their objective cognitive state evolution to fully tailor instructions to their abilities (Gerjets et al., 2014; Sweller, 2020). One solution to this problem is the real-time measurement of cognitive load through the electrical activity of the brain using an Electroencephalogram (EEG)-based Brain-Computer Interface (BCI) system.

BCIs facilitate direct communication between the brain and computers by converting the brain’s electrical signals into computer commands (Gao et al., 2021; Lotte et al., 2018; Zander and Kothe, 2011). Initially created to assist individuals with disabilities in controlling external devices (Värbu et al., 2022), BCIs now extend to passive systems that monitor cognitive states, such as attention, fatigue, engagement, and cognitive load (Zander and Kothe, 2011), enhancing cognitive functions through self-regulation and neurofeedback (Birbaumer et al., 2009). These systems provide feedback based on brain activity changes, forming a closed biocybernetic loop (Krol and Zander, 2017). BCIs potentially offer tailored learning experiences in education by adjusting educational content based on real-time brain activity analysis.

Thus, the purpose of this study is to investigate whether the use of a neuroadaptive interface would provide an optimal learning experience and increase learning gains with the following research question: “*Does adapting the pace of information presentation to the learner’s real-time cognitive load using an EEG-based passive BCI enhance the learning experience?*.” Specifically, we developed an EEG-based BCI system that adapts the speed of information presentation on the Interactive User Interface (IUI) according to the real-time cognitive load of the learners. We created a memory-based learning task following the ZPD theory to test our BCI system. The dynamic adaptive measures of our BCI are designed to help learners manage their cognitive load and stay within their ZPD for an optimal learning experience. We define an optimal learning experience as the intersection of increased learning gains, self-perceived cognitive absorption and satisfaction, and reduced self-perceived cognitive workload.

Furthermore, the limited research on the use of BCI in education fails to account for the impact of motivation on adaptation. While it is established that motivation influences the cognitive effort invested in a learning task (Paas et al., 2005), there is a dearth of information on this topic in the context of BCI-based learning. We also aim to investigate if the addition of a motivational factor while using the BCI would enhance the learning experience with the following research question: “*To what extent is motivation a necessary condition for effective BCI adaptation?*”

To the best of our knowledge, our study is the first of its kind, combining a novel BCI system and a memorization-based learning task developed following the ZPD theory. Our research stands out as very few papers study neuroadaptive interfaces in a CBL context. Existing papers on the topic have used BCIs to monitor different cognitive states (Andreessen et al., 2021; Marchesi and Riccò, 2013; Zammouri et al., 2018; Zhou et al., 2017b), detect and react to error potentials (Buttfield et al., 2006; Spüler et al., 2012), to adjust different interface parameters, such as task difficulty or content type (Eldenfria and Al-Samarraie, 2019) or provide user cognitive state feedback (Verkijika and De Wet, 2015). In contrast, we employ a BCI system that uses real-time data to estimate and classify cognitive load to adapt the speed of information presentation on the interface.

The remainder of this manuscript is organized as follows. We first present related literature and the development of the hypotheses. We then present the materials and methods used in this study, including core aspects of developing our BCI system. We then present our data analysis and study results. Findings are interpreted within the discussion section. Finally, the article concludes with a short conclusion encompassing limitations and future research avenues.

## 2 Related work

### 2.1 The need for individual learning paces within the zone of proximal development

The ZPD theory suggests that all students have different learning needs and abilities, therefore different ZPDs. Thus, within ZPD, each student assimilates and processes new information or acquires abilities differently; some learners need more time and effort than others to learn successfully (Hedegaard, 2012).

Studies have shown that in order to increase information retention and promote optimal learning experiences, learning pace must be adjusted and personalized to each student (Najjar, 1996; O'Byrne and Pytash, 2015; Shemshack and Spector, 2020). For example, Hasler et al. (2007) investigated the differences between imposed system-paced and personalized learner-paced groups on primary school students. They found that self-perceived cognitive load was lower and test performance was higher when students used the learner-paced system, which suggests that allowing students to control their own learning pace may improve learning outcomes. Andreessen et al. (2021) also investigated the effect of text difficulty and text presentation speed in a reading task on self-perceived mental workload. Some texts, varying in difficulty, were presented at the reader’s pace, and some were presented at a 40% faster pace. Cognitive load predicted values and subjective mental workload experienced were significantly higher when learners read at a fast-imposed speed.

In short, these studies demonstrate the importance of adapting learning tasks, educational content, and instructional strategies to each learner’s learning pace to promote an optimal learning experience. These studies also suggest that CBL environments facilitate the personalization of learning methods and processes.

### 2.2 Personalizing computer-based learning environments

CBL has created new opportunities for personalized learning in the digital era. Personalizing learning through CBL can help address each learner’s diverse learning needs by adapting instructional materials to their learning pace and progress, which can help optimize the ratio of challenge to support explained by the ZPD to suit each learner.

Recent CBL environment studies rely on users’ personal and learning data to create algorithms that personalize the learning experience. For example, Xiao et al. (2018) developed a personalized system that recommends learning materials based on an algorithm combining the student’s learning path and interests. Results from the pilot testing indicated that their system increased the learners’ learning outcomes and satisfaction levels. El-Sabagh (2021) developed an online learning environment that suggests content based on the student’s learning style and adapts the modules based on behavioral data (learning activities, errors, navigation). They found that the participants who used the adaptive learning environment had better overall performance scores and higher reported engagement levels than those who did not. Ku and Sullivan (2002) also developed an adaptive learning system that adapts mathematical questions based on the learner’s interests (favorite foods, sports, etc.) and discovered that the system enhanced the students’ learning achievement and positively affected their learning attitude. Finally, Tekin et al. (2015) developed *eTutor*, a personalized online learning platform, that learns the best order in which to deliver instructional materials with an algorithm based on the learner’s preferences and needs, and uses their feedback input on previously presented instructional contents (such as exam scores and time spent on a course) to adapt the educational material. They found that their system improved performance on assessments and achieved significant savings in the amount of time that students spent learning.

These studies have demonstrated that adaptive CBL environments can positively impact the learner’s learning experience. However, their assessment methods do not account for the learner’s real-time cognitive load, which can substantially affect learning effectiveness and efficiency (Sweller, 1988, 2020).

### 2.3 Cognitive load and measurement approaches

The CLT postulates the importance of minimizing the mental effort associated with the processing of the instructional design or the learning environment (Curum and Khedo, 2021; DeLeeuw and Mayer, 2008) that is unrelated to the learning itself (Extraneous Load) and managing the level of complexity of both the learning material and the learning task itself (also known as Intrinsic Load) (Sweller, 2010), in order to reduce the overall cognitive load and thereby optimize the use of working memory resources [known as Germane Load (Debue and van de Leemput, 2014) or Germane Processing (Sweller et al., 2019)]. We refer to this sweet spot as the “Goldilocks Zone” (Karran et al., 2019), where the overall cognitive load is optimized to enhance the learning process and increase performance.

ZPD and cognitive load are closely linked concerning the personalization and optimization of learning experiences. Learning tasks that align with a student’s ZPD are less likely to overwhelm them, helping to reduce their Extraneous Load (Schnotz and Kürschner, 2007). In addition, instruction tailored to a learner’s ZPD facilitates the learning and minimizes their Intrinsic Load (Schnotz and Kürschner, 2007). Thus, the ZPD makes it possible to evaluate the learner’s cognitive abilities to avoid cognitive overload and underload, leading to poor learning outcomes (Paas et al., 2004).

It is essential to measure and assess the cognitive load of learners to adjust their learning environments and enhance their learning experiences and outcomes. Today, self-reported measures remain the most used method to measure cognitive load in the research and development of various educational technology tools as they offer the learners’ perspectives on their experience (Anmarkrud et al., 2019; Brunken et al., 2003; Mutlu-Bayraktar et al., 2019). However, they cannot objectively and precisely capture and quantify the amount of mental work expended during the learning process (Mutlu-Bayraktar et al., 2019). Self-perceived measures also rely on the learners’ subjective awareness and perceptions, which involve a deeper reflection and thought process about their learning experience (Ayres, 2006). Learners must reflect upon their learning experience, considering the cognitive effort and mental processes involved, influenced by their level of metacognitive awareness. While subjective measures offer insights into the perception of cognitive load, they do not fully capture the learner’s evolving cognitive state, which is necessary to tailor instructions to their abilities. Utilizing physiological measurement tools such as eye movement data, hormone levels, heart rate variability, and brain activity (Riedl and Léger, 2016) can provide a more precise, reliable, valid and complementary continuous cognitive load assessment (Brunken et al., 2003).

Among the various tools available for brain imaging, EEG is one of the most used due to its non-invasive, cost-effective, convenient, accessible features and high temporal resolution (Abiri et al., 2019; Antonenko et al., 2010). EEG measures voltage fluctuations in cortical activity, which can be used to assess and infer mental states. Different cognitive processes are associated with variations in brainwave patterns, specifically frequency, amplitude, synchronization between neural networks, and Event-Related Potentials (ERPs) in response to stimuli (Riedl and Léger, 2016). Previous research on cognitive load suggests that theta (*θ*, 4–7 Hz) and alpha (*α*, 8–12 Hz) oscillations are associated with task difficulty, with alpha activity becoming desynchronized (or decreased) and theta activity becoming synchronized (or increased) as task difficulty increases (Antonenko et al., 2010; Gevins and Smith, 2003; Klimesch, 1999; Stipacek et al., 2003). Dynamic changes in alpha activity would mainly occur in the brain’s posterior regions, while changes in theta activity would mainly occur in the brain’s frontal regions (Cavanagh and Frank, 2014; Tuladhar et al., 2007). Prior research used a visuospatial working memory task to explore whether variations in brain activity synchronization within and between the frontal and parietal regions stem from differing central executive demands (Klimesch et al., 2005). The findings indicated that activity synchronization between these areas’ mirrors working memory’s executive functions: increased executive load leads to reduced anterior coupling in the upper alpha range (10–12 Hz) and heightened theta synchronization between frontal and parietal regions.

### 2.4 Brain-computer interfaces

BCIs enable direct brain-to-machine communication and interaction, allowing users to manipulate and engage with technology (Gao et al., 2021; Lotte et al., 2018; Zander and Kothe, 2011). BCI research has gained much popularity in recent years due to its potential medical applications (Gu et al., 2021), such as for neurorehabilitation in brain injury, motor disability and neurodegenerative diseases (Abiri et al., 2019; Chaudhary et al., 2016; Daly and Wolpaw, 2008; Pels et al., 2019; Vansteensel et al., 2023), detection and control of seizures (Liang et al., 2010; Maksimenko et al., 2017), and improvement of sleep quality and automatic sleep stages detection (Papalambros et al., 2017; Phan et al., 2019). Several studies have also looked at non-clinical applications, such as video games (Ahn et al., 2014; Kerous et al., 2018; Laar et al., 2013; Labonte-Lemoyne et al., 2018; Lalor et al., 2005; Lécuyer et al., 2008), marketing and advertisement (Bonaci et al., 2015; Mashrur et al., 2022; Tadson et al., 2023), neuroergonomics and smart environments (Carabalona et al., 2012; Kosmyna et al., 2016; Lin et al., 2014; Tang et al., 2018), and work monitoring and safety (Aricò et al., 2016; Demazure et al., 2019; Demazure et al., 2021; Karran et al., 2019; Roy et al., 2013; Venthur et al., 2010). A BCI is classified as a neuroadaptive interface (Riedl et al., 2014) when real-time adaptations occur on an interface presented on a computer.

Most BCIs use EEG to acquire brain signals (Lotte et al., 2018). Depending on the type of research conducted, EEG-based BCIs can be invasive (with electrodes placed directly on the surface of the brain) or non-invasive (with electrodes placed on the scalp of the subject) (Abiri et al., 2019). Invasive EEG-based BCIs have the advantage of directly measuring higher-quality brain signals, reducing external interference (Daly and Wolpaw, 2008). However, they require surgery to insert and remove the electrodes, exposing patients to several potential complications (Daly and Wolpaw, 2008; Värbu et al., 2022). In contrast, non-invasive EEG-based BCIs measure brain activity using electrodes placed on the scalp. The major drawback is that these electrodes are subject to several factors that affect the quality of the recorded signal, such as external noise, a weaker electrical signal, and even the physical movements of the subject (Padfield et al., 2019). Nevertheless, non-invasive EEG-based BCIs remain more popular due to their noninvasiveness while providing high temporal resolution and a low cost (Abiri et al., 2019; Cohen, 2017; Dimoka et al., 2012; Lotte et al., 2018; Värbu et al., 2022).

In general, brain signals are typically first acquired with an EEG (Lotte et al., 2018), which are then processed through a series of steps, including data preprocessing, feature extraction and signal classification (Padfield et al., 2019), before finally being interpreted by the BCI and used for its purpose (Abiri et al., 2019; Lotte et al., 2018).

There are three main BCI paradigms: *active*, *reactive*, and *passive* (Table 1). Active paradigms allow users to directly control the system by deliberately controlling their brain activity (Ahn et al., 2014; Angrisani et al., 2021; Zander and Kothe, 2011; Zander et al., 2009). For instance, users can employ mental imagery to imagine motor movements, allowing the system to replicate the intended action on a screen or with an external device, such as a mechanical arm (Steinert et al., 2019). In reactive paradigms, specific brain activity initiates predetermined actions from the system in response to external stimuli (Ahn et al., 2014; Wang et al., 2019; Zander and Kothe, 2011). Brain reactivity measured following external stimuli is associated with a specific command from the system, making this type of BCI very specific and efficient (Dehais et al., 2022). For example, Chen et al. (2017) used Steady-State Visual-Evoked Potentials (SSVEP) to develop a reactive BCI in a visual navigation task. SSVEPs were detected by the BCI when participants were looking at the sides of a flickering square in the middle of the screen, which allowed them to control the direction of the cursor. Finally, in a passive paradigm, brain activity is continuously monitored to differentiate or quantify mental states without user control, providing feedback as a system response. For example, Karran et al. (2019) developed an EEG-based passive BCI to measure and monitor users’ sustained attention in a long-duration business task. The system’s feedback consisted of countermeasures in the form of color gradients representing the participant’s sustained attention level and alerts when sustained attention was low as forms of system feedback to maintain sustained attention at an optimal level and improve performance.

**Table 1:** Overview of brain-computer interface (BCI) paradigms: control types, user involvement, applications and advantages.

	Control type	User involvement	Common applications	Advantages
Active BCI	User-driven, conscious control of brain activity	High: deliberate modulation of brain signals by the user (e.g., motor imagery).	Neuroprosthetics, motor control (e.g., robotic arm).	Fine-tuned control for specific tasks, useful for disabled users needing direct control.
Reactive BCI	Stimulus-driven, system reacts to external stimuli.	Medium: passive response to external stimuli (e.g., Steady-State Visual Evoked Potentials (SSVEP), P300).	Speller systems, attention-based interfaces.	Efficient, system commands are linked to specific brain responses to stimuli.
Passive BCI	System-driven, monitors brain states without user control.	Low: no direct user control; continuous monitoring of spontaneous brain activity.	Cognitive workload assessment, fatigue monitoring, adaptive systems.	Non-intrusive, ideal for monitoring and real-time adaptation to mental states.

Passive BCIs have garnered significant attention recently, especially for implementing closed-loop adaptations (Krol and Zander, 2017). In a passive closed-loop BCI, real-time brain activity and adaptive system actions continuously influence each other as part of a biocybernetics loop (Ahn et al., 2014; Krol and Zander, 2017; Pope et al., 1995; Roy et al., 2013; Zander and Kothe, 2011). This dynamic cycle begins when an assessed brain state triggers an adaptive response from the system. The system then provides feedback or adjusts the content to alter the current brain state, and so forth (Krol and Zander, 2017). The aforementioned study by Karran et al. (2019) is an example of a closed-loop BCI, as the system continuously monitors sustained attention and provides feedback according to the level measured to influence the user to increase their sustained attention. This biocybernetics loop continued until the end of the experiment.

### 2.5 Brain-computer interfaces in educational contexts

The application of BCIs in diverse settings demonstrates their innovative potential to enhance learning outcomes and empower learners through novel interactions with educational content. However, research on using BCIs in educational contexts is limited and inconsistent (Xia et al., 2023). Previous studies have primarily employed passive BCIs to achieve mental state assessments of users as they learn and interact with educational interfaces, subsequently personalizing learning according to the data collected (Krol and Zander, 2017). For example, Apicella et al. (2022) developed a wearable EEG-based system to detect and classify students’ cognitive and emotional engagement during learning tasks, leveraging brain signals to optimize adaptive learning platforms in real-time. Engagement was measured using EEG signal analysis through a Filter Bank and Common Spatial Pattern (CSP) method, followed by classification with a Support Vector Machine (SVM). The task involved a Continuous Performance Test (CPT) to modulate cognitive engagement, while emotional engagement was influenced by background music and social feedback. The system achieved classification accuracies of 76.9% for cognitive and 76.7% for emotional engagement. In addition, previous research on cognitive load and adaptive educational interfaces has mainly focused on the complexity of the educational material and the instructional guidance presented to the learner (Kalyuga and Liu, 2015; Mutlu-Bayraktar et al., 2019; Petko et al., 2020). These gaps in the literature have recently prompted researchers to investigate the transformative potential of passive closed-loop BCIs in learning contexts.

For instance, Yuksel et al. (2016) created a passive closed-loop BCI called Brain Automated Chorales (BACh), which adjusts the difficulty level of piano learning material according to cognitive workload measurements obtained through functional near-infrared spectroscopy (fNIRS). Adaptive measures of the system depended on learners’ cognitive workload throughout both the training and learning tasks, which were classified using a machine learning algorithm. The results suggest that the learners’ playing speed and performance accuracy improved when learning piano with the BACh system. Additionally, the learners reported a better learning experience with the system and noted that difficulty levels were appropriately adjusted. Additionally, Walter et al. (2017) designed a closed-loop EEG-based BCI that measures cognitive workload in real-time to adapt the difficulty of arithmetic problems presented in an online learning environment. Cognitive workload classifications were separated into three difficulty levels based on workload state predictions derived from a pre-trained regression model to determine the optimal range of cognitive workload for learning. Their findings demonstrated that participants who completed the experiment with the adaptive instructions achieved greater learning gains than those who completed the experiment without adaptivity. However, this difference was not statistically significant. Finally, Kosmyna and Maes (2019) created AttentivU, an EEG-based passive closed-loop BCI that measures engagement in real-time and triggers haptic feedback (vibrations from a scarf worn by the learner) when a drop in engagement is detected. The system used the engagement index proposed by Pope et al. (1995), which calculated the average power of theta, beta and alpha frequency components derived from Power Spectral Density to return a smoothed engagement index every 15 s. The two studies conducted with AttentivU yielded results indicating that haptic biofeedback driven by BCI redirected learners’ engagement to the task, resulting in enhanced performance on comprehension tests. These studies demonstrate the feasibility of closed-loop BCI systems within educational contexts to adapt and personalize learning to each learner.

The aim of the current study is to investigate the effects of an EEG-based passive closed-loop BCI on the learning experience in a memory-based learning task and contribute to the literature regarding the effects of closed-loop passive BCI on learning outcomes.

### 2.6 Hypotheses development

**Figure 1:**
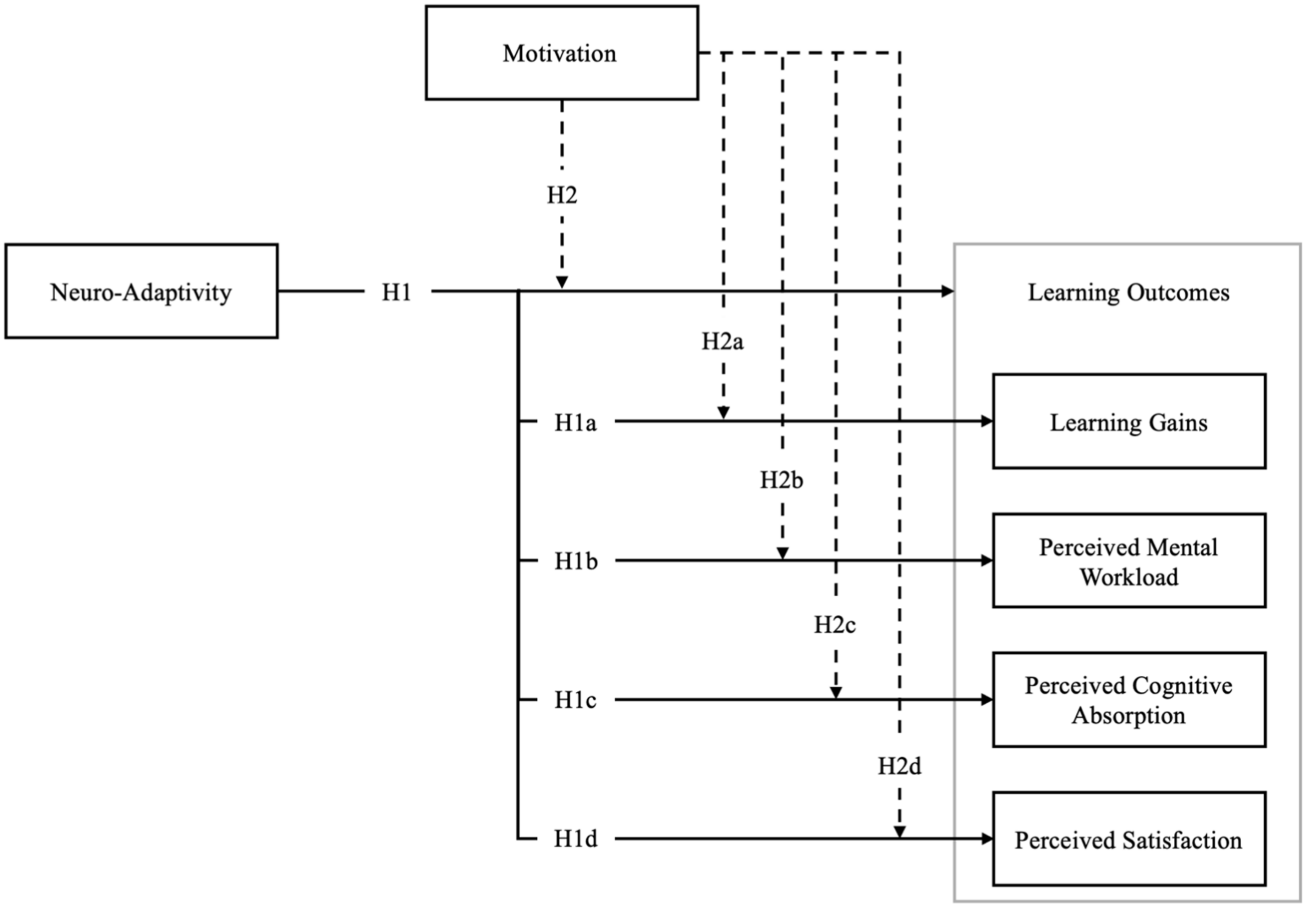
Conceptual framework illustrating the effects of neuro-adaptivity and motivation on learning outcomes.

Our study aims to answer the following research question: “*Does adapting the pace of information presentation to the learner’s real-time cognitive load using an EEG-based passive BCI enhance the learning experience?*.” We hypothesize that (H1) “*neuro-adaptivity enhances the learning experience compared to the absence of neuro-adaptivity*” (Figure 1). This study defines the learning experience as a combination of objective and subjective measures of cognitive load and emotional state, specifically focusing on learning gains, perceived mental workload, perceived cognitive absorption, and satisfaction.

Learning gains in this context represent an objective measure of the knowledge learned and memorized throughout the experimental task, allowing an assessment of the impact of the BCI on learning. Prior research suggests aligning learning speed with cognitive load can enhance efficiency and effectiveness (Petko et al., 2020). We propose that neuro-adaptivity leads to greater learning gains by optimizing the learning pace to the learner’s cognitive load. Thus, we hypothesize (H1a) that *neuro-adaptivity leads to more significant learning gains compared to the absence of neuro-adaptivity* (Figure 1).

Additionally, understanding how learners perceive and estimate their mental workload while working with and without the BCI, is necessary for evaluating the learning experience. Perceived mental workload refers to the perceived mental effort required to complete the learning task and its impact on the experience (Hancock and Meshkati, 1988), where higher perceived mental workload translates into a less optimal learning experience (Sweller, 1994). Therefore, we hypothesize that (H1b) “*neuro-adaptivity reduces perceived mental workload compared to the absence of neuro-adaptivity”* (Figure 1).

Derived from Csikszentmihalyi’s theory of flow (Csikszentmihalyi, 1975; Csikszentmihalyi, 2014), cognitive absorption is described as a state of total immersion when performing a task, characterized by high levels of engagement and focus (Agarwal and Karahanna, 2000). Previous studies have shown that higher levels of cognitive absorption while completing CBL tasks lead to higher satisfaction levels and better-perceived ease of use and usefulness of the learning tool (Saadé and Bahli, 2005; Salimon et al., 2021). Therefore, we hypothesize that (H1c) “*neuro-adaptivity generates a higher self-perceived cognitive absorption level than the absence of neuro-adaptivity”* (Figure 1).

Learner satisfaction reflects the degree to which learners feel engaged, satisfied, and fulfilled with their learning experiences (Martin and Bolliger, 2022; Wickersham and McGee, 2008). Previous research has shown that learner satisfaction leads to better learning outcomes (Martin and Bolliger, 2022). Therefore, we hypothesize (H1d) that *“neuro-adaptivity generates a higher level of perceived satisfaction with the learning experience compared to the absence of neuro-adaptivity”* (Figure 1).

Furthermore, we aim to examine the role of motivation, both *intrinsic* and *extrinsic*, in the learning experience during BCI utilization. Numerous studies have demonstrated the importance of motivation in achieving academic success, notably in CBL environments (Hu et al., 2016; Lepper and Malone, 2021; Nikou and Economides, 2016). To aid in this examination, we ask the following research question: “*To what extent is motivation a necessary condition for effective BCI adaptation?*”

In general, learners are more likely to be actively engaged and motivated when the learning experiences provided are specific to their ZPD (Shabani et al., 2010; Vygotsky and Cole, 1978). Self-determination theory (SDT) investigates the motivations of individuals in varying social contexts and situations. It identifies two types of motivation: intrinsic and extrinsic (Ryan and Deci, 2000a,b). When learners are intrinsically motivated, they will learn naturally, usually with interest and enjoyment, because of the benefits that the subject matter can bring (Ryan and Deci, 2000a,b). Whereas, extrinsic motivation occurs when learners compel themselves to learn to obtain a reward or avoid consequences (Ryan and Deci, 2000a,b). Extrinsic incentives such as money or prizes have been demonstrated to enhance learning performance (Schildberg-Hörisch and Wagner, 2020) by improving attention (Anderson, 2016; Small et al., 2005), effort (Schwab and Somerville, 2022), and working memory (Wimmer and Poldrack, 2022) and can motivate students to remain interested, engaged, and dedicated to their learning, resulting in greater learning outcomes (Festinger et al., 2009; Gong et al., 2021; Rousu et al., 2015).

These findings suggest that extrinsic motivators can support intrinsic motivation. Therefore, we will utilize extrinsic motivation in the form of a financial incentive to help answer our research question. We hypothesize that (H2) *motivation moderates the effect of neuroadaptation by increasing its effectiveness and perception of an optimal learning environment when compared to the neuro-adaptive interface alone* (Figure 1). More precisely, we hypothesize that (H2a) *adding motivation to neuro-adaptivity helps to achieve greater learning gains compared to neuro-adaptivity alone*; (H2b) *adding motivation to neuro-adaptivity reduces perceived mental workload compared to neuro-adaptivity alone*; (H2c) *adding motivation to neuro-adaptivity generates a higher level of perceived cognitive absorption than neuro-adaptivity alone*; (H2d) *adding motivation to neuro-adaptivity generates a higher level of self-perceived satisfaction of the learning experience compared to neuro-adaptivity alone* (Figure 1).

## 3 Materials and methods

### 3.1 Participants

Fifty-five participants participated in our study (27 ± 7.92 years old, 28 female), 36 university students, 19 took online classes or training regularly for professional or personal reasons. All participants were recruited by e-mail from our institution’s panel database. Participants were included based on age greater than 18 years old, normal or corrected-to-normal vision, having no history of neurological conditions, right-handedness, fluency in the French language, and high computer proficiency. Handedness was validated before the experiment with the Edinburgh Handedness Inventory (Caplan and Mendoza, 2011), and all other inclusion criteria were validated through the screening questionnaire. Participants signed a consent form before completing the study and were informed they could leave it anytime. Participants were compensated 100$ (CAD) for their participation. Our institution’s ethics committee approved the study under certificate 2023–5,071.

### 3.2 Experimental design

#### 3.2.1 Experimental conditions

We utilized a 3 × 2 (type of adaptation x motivation) between-subject design. Participants were randomly assigned to a group prior to data collection and kept unaware of experimental factors. In the current study, conditions refer to type of Interactive User Interface (IUI): Control (C) no adaptivity (*n* = 17), stimuli are presented at predefined intervals; Adaptive (A) without motivation (*n* = 22), stimuli are presented at variable speeds based on a classification of user cognitive load; Adaptive (AM) with motivation (*n* = 16), stimuli are presented at variable speeds based on a classification of user cognitive load in the presence of financial motivation. For the AM group to provide extrinsic motivation, participants were informed that better overall task performance resulted in more entries in a $200 Visa prepaid gift card prize draw. To conform with ethical principles, all participants, regardless of experimental condition, received the same number of entries for the prize draw when the study concluded.

#### 3.2.2 Phase one: calibration

**Figure 2:**
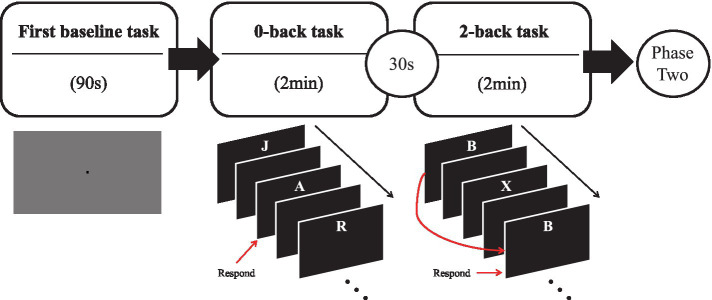
Schematic representation of the n-back task used in the calibration task (phase 1).

As illustrated in Figure 2, the first part of the calibration phase consisted of a 90s baseline task used for post-hoc analyses, where participants had to stare at a black square in the middle of a grey screen. The second part of the calibration phase consisted of an *n*-back task to estimate personal threshold values of high and low cognitive load. These thresholds were then integrated into the BCI model to personalize the classifier’s thresholds and limits (*Sections 3.2.4* and *3.3.2*). This task was performed regardless of condition.

The *n*-back task was selected due to its popularity for manipulating memory load, which can serve as a proxy for cognitive load (Brouwer et al., 2012; Grimes et al., 2008; Wang et al., 2016) and its similarity to the learning task, which requires memory and recall of visual stimuli. In the *n*-back task, participants must assess whether each stimulus in a sequence corresponds to the stimulus presented *n* items earlier (Hogervorst et al., 2014). As *n* increases, the *n*-back task becomes more challenging, requiring more cognitive resources. A four-minute n-back task was administered in two parts: a 2-min 0-back task to assess low cognitive load and a 2-min 2-back task to assess high cognitive load, separated by a short break of 30 s. Each stimulus (letter) was presented for one second, followed by a two-second intertrial interval for both tasks, resulting in a new letter being presented every three seconds, totaling 40 iterations.

#### 3.2.3 Phase two: learning task

One of the most frequent learning tasks in higher education involves memorizing course material for exams and practical applications due to the sheer quantity of information that must be learned within a limited time frame. To test our hypotheses, we adapted an existing constellation memorization learning task (Riopel et al., 2017).

Star constellations were chosen as the learning topic for two reasons. First, university students typically possess low prior knowledge about the subject. Second, even the most knowledgeable individuals easily encounter unfamiliar material. The task required participants to select the correct name of a constellation from three options associated with an image of one of the 88 constellations. The purpose was to examine the learning, forgetting, and spacing curves in online learning. This allowed us to design a valid task that could promote learning while inducing changes in cognitive load.

**Figure 3:**
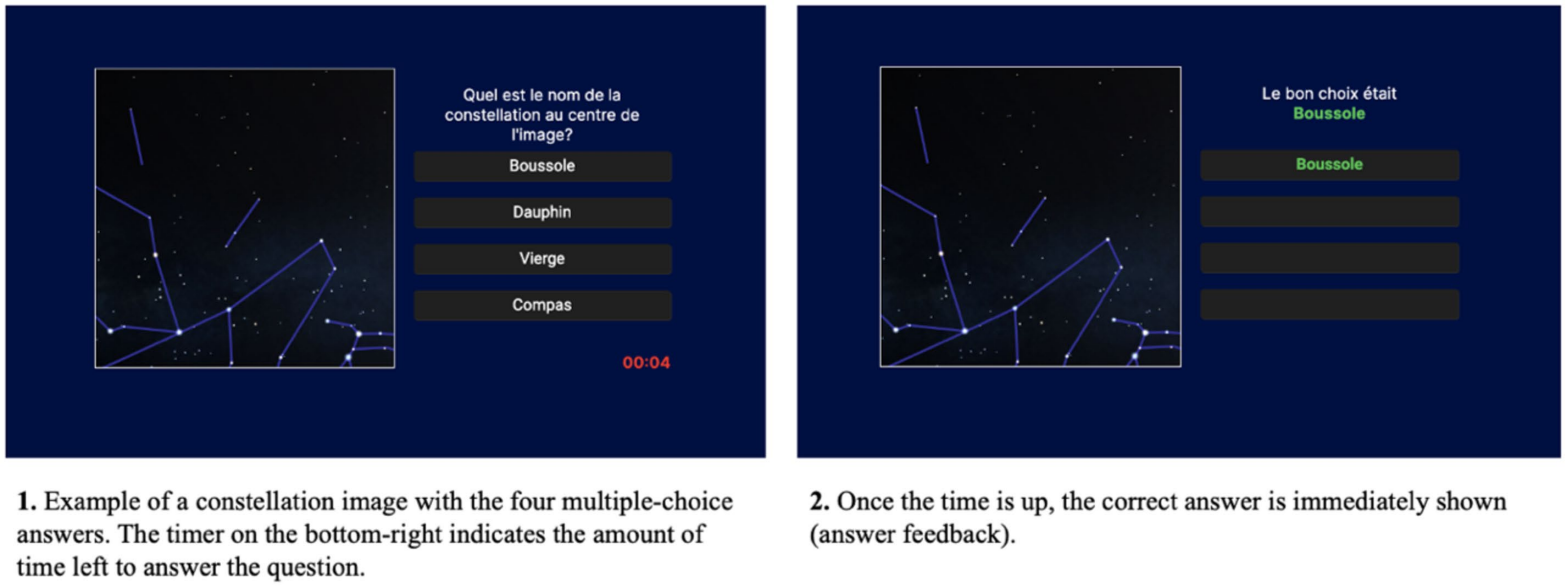
Example of a constellation from the learning experiment, presented on the interface.

As indicated in Figure 3, participants were instructed to memorize as many constellations as possible by associating the presented constellation image with its corresponding name from a choice of four multiple-choice answers. The correct answer (feedback) was displayed after each question, regardless of whether it was answered correctly or not. Previous research has indicated that providing the correct answer to a question, irrespective of whether it was answered correctly, is essential in enhancing the retention of information and avoiding future mistakes (Butler et al., 2008; Kulhavy, 1977). The instructions remained the same throughout the learning task, which contained four blocks (i.e., trials) of questions, separated by short breaks of 30 s (see *Section 3.2.5*). Participants were required to memorize 32 constellations, each presented twice per block. The sequence of constellation presentation was pre-randomized before data collection and remained the same for all participants. However, the correct answer’s position among the four multiple-choice options and the three incorrect constellation names were randomized.

#### 3.2.4 Model of adaptivity and cognitive load classifications

The model of adaptivity used in our study was adapted from Karran et al. (2019) who conceived of an adaptive model of sustained attention, in which two thresholds denote the chance of failure, an upper soft limit beyond which chances of failure increase, and a lower hard limit beyond which failure is certain, the model is such that adaptive countermeasures are provided to keep a user of the BCI within the upper and lower bounds in what they term the “goldilocks” zone, i.e., neither to high nor too low. We chose this model because it was easily adapted to replace sustained attention with cognitive load, while keeping all thresholds the same. In the current study, we inverted the limits such that the upper limit represents cognitive overload and the certainty of failure, and the lower limit represents to little cognitive load and an increased chance of failure through inattention or boredom. The “goldilocks” zone represents the ZPD, which promotes an optimal cognitive load level, which is not too high or too low, through fluid and dynamic adaptations to enhance learning gains over time.

EEG analysis between the 0-back and the 2-back tasks controlling the False Discovery Rate (FQR, q = 0.05) of 17 pre-tests demonstrated a significant decrease in alpha-band activity within the parietal, occipital, and right temporal regions. However, the same analyses with a Bonferroni correction suggested a significant reduction in alpha-band (*α*) activity at the P7 electrode. Consequently, we exclusively used the P7 electrode when computing the cognitive load index, which aligns with the current literature (see *Section 2.3*). We used an index based on average alpha-band power in the parietal cortex (electrode P7) during 6-s sliding windows with no overlap to calculate the cognitive load.


$$CL_{current}=P_{\alpha ,i}$$

Where $CL_{current}$ represents a new real-time index value, i.e., the current cognitive load level, by calculating the alpha-band power activity during the *i*th 6-s sliding window, denoted by $P_{\alpha ,i}$ .

As described in *Section 3.2.2*, the *n*-back task was used to determine baseline cognitive load thresholds. Specifically, cognitive load averages for the 0-back and 2-back tasks were calculated separately using the cognitive load index. This resulted in the creation of two thresholds, which represent “low average” and “high average” cognitive load. In addition, the average cognitive load for the entire *n*-back task was calculated.


$${\bar{CL}}_{0back}\;\mathrm{and}\;{\bar{\mathrm{CL}}}_{2back}=\frac{\sum_{i=1}^{N}CL_{current}}{N}$$

$${\bar{CL}}_{nback}=\frac{CL_{\;0back}+CL_{\;2back}}{2}$$

Where ${\bar{CL}}_{0back}$ and ${\bar{CL}}_{2back}$ denote the average cognitive load for the 0-back or the 2-back task, respectively. N represents the total number of 6-s sliding windows during the task, used to calculate the average of the task. $CL_{current}$ represents the real-time cognitive load level, i.e., a new real-time index value, calculated with the cognitive load index. Finally, the average cognitive load level is calculated using the 0-back and 2-back task thresholds, denoted by ${\bar{CL}}_{nback}$ .

The real-time index values were stabilized during the learning experiment using a 60-s sliding window that dynamically adjusted the average cognitive load over time. In other words, decisions on cognitive load classifications were made every 6 s based on the index compared with a moving average of the previous 60 s or the last 10 data points. This ensured that the classification would adjust to changes in the user’s cognitive state throughout the experiment. Additionally, analysis of the 17 pre-tests indicated a 125% increase in the amplitude of the alpha-band signal during the learning task compared with the *n*-back task. These results suggest that the thresholds should be 1.25 times higher than the average values obtained in *n*-back. Therefore, the resulting cognitive load value exceeding the “high average” threshold would result in a classification as “2” in the BCI system, indicating a high cognitive load level. Conversely, a resulting cognitive load value below the “low average” threshold would classify as “0” in the BCI system, indicating a low cognitive load level. Finally, when the resulting cognitive load value fell between the “high average” and “low average” thresholds, it would be converted to a “1” classifier in the BCI system, indicating an optimal level of cognitive load.


$$MA_{i}=\frac{\sum_{j=i-9}^{i}CL_{current}}{10}$$

$$Class0=MA_{i}\times \left(\frac{{\bar{CL}}_{0back}}{{\bar{CL}}_{nback}}\right)\times 1.25$$

$$Class2=MA_{i}\times \left(\frac{{\bar{CL}}_{2back}}{{\bar{CL}}_{nback}}\right)\times 1.25$$

Where $CL_{current}$ represents the real-time cognitive load value, calculated with the index. Therefore, $MA_{i}$ represents the moving average of the last ten cognitive load values at time *i*. The factor of 1.25 represents the threshold adjustment according to the results obtained in the pre-tests.

#### 3.2.5 Adaptive rules of the interface and specifications

**Figure 4:**
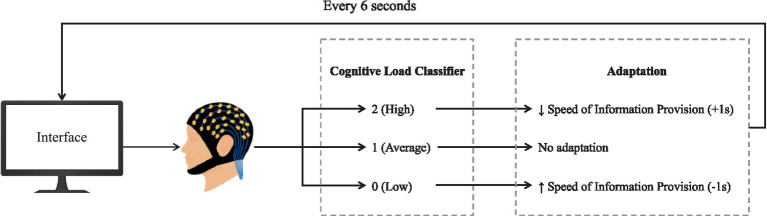
Adaptive rules of the BCI system implemented in the experiment.

During the learning task, the adaptive Intelligent User Interface (IUI) modulated the information delivery speed (see Figure 4). Specifically, upon receiving high cognitive load classifications (“2”), the interface slowed information delivery, affording participants extended time for question response and correct answer processing. Conversely, low cognitive load classifications (“0”) triggered an increase in delivery speed, reducing the response and correct answer display time. No adjustment was made for classifications of average cognitive load (“1”), indicating optimal cognitive load. Following ZPD theory, we posited that these time adaptations would allow the learner to remain in their ZPD, leading to better learning outcomes. Thus, the baseline time window for displaying constellation questions and feedback was 5 s. Based on pre-test results, adjustments were made in 1-s increments within a 3 to 8-s range per item. Pre-tests revealed that presentations over 8 s diminished response efficiency and significantly lowered engagement, focus, and interest, aligning with existing research findings (Beck, 2005; Chipchase et al., 2017). The minimum time was set at 3 s to prevent the BCI system from getting confused between the brain’s processing of new information and high cognitive load levels (Anderson et al., 2011; Rosso et al., 2001; Vijayalakshmi et al., 2015). Finally, the constellation question and the feedback were presented for the same duration to ensure adequate time for participants to respond and process the correct answer.

**Figure 5:**
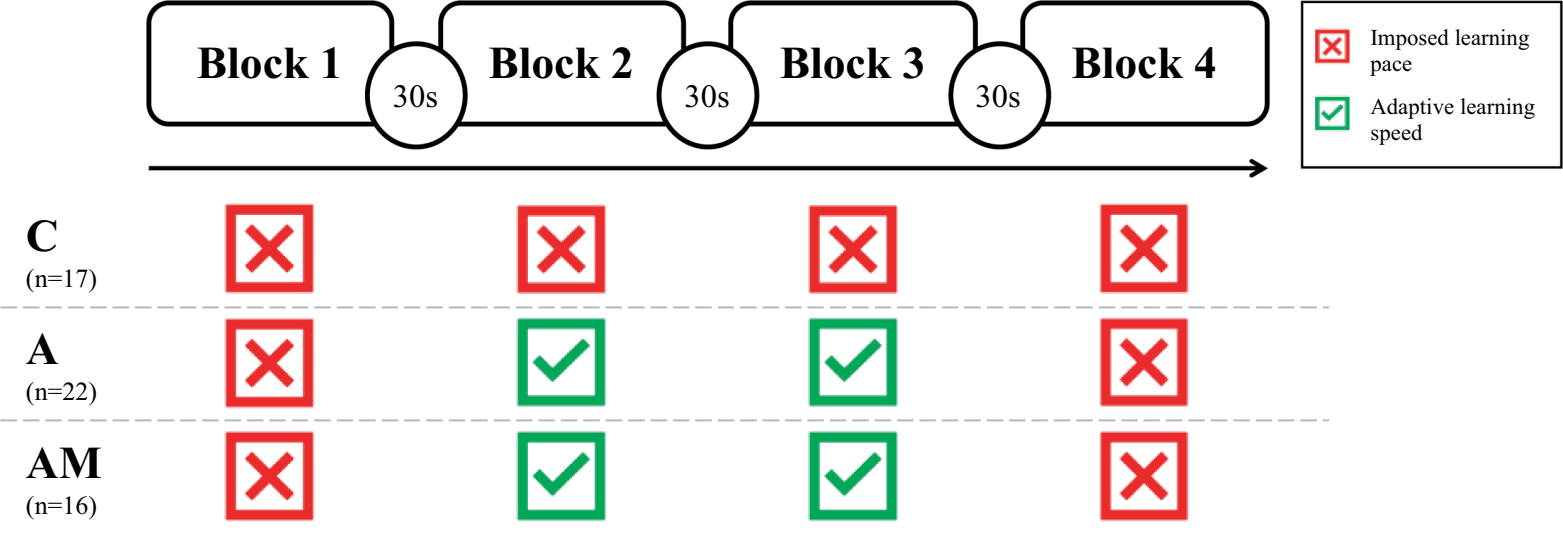
The learning task: adaptivity of each block for each group.

Figure 5 illustrates the learning task, which was structured into four blocks, interspersed with 30-s intervals. In the C group, question and feedback pacing remained constant across all blocks, adhering to a 5-s baseline. For the A and AM groups using the adaptive IUI, information delivery rates in the second and third blocks were modulated based on cognitive load classifications from the BCI; no adaptation was applied in the first and last blocks to assess the effect. To facilitate participant re-engagement post-breaks, the initial 30 s (or first three constellations) of the adaptive blocks maintained the baseline delivery speed of 5 s for both questions and feedback.

No adaptation occurred while a constellation and its correct answer were displayed. This way, if a high or low cognitive load classification were received during this period, any change in the speed of information provision would only affect the next constellation to avoid confusing the learner. To prevent unnecessary stress during short response times and loss of interest or focus during longer response times, a countdown timer was clearly displayed below the multiple choices to assist participants in managing their expectations (Ghafurian et al., 2020). Finally, neither correct nor incorrect answers influenced the speed of information presentation, only the cognitive load classification.

### 3.3 Apparatus

#### 3.3.1 Interactive user interface

The constellation learning IUI was presented to the participants on a 22-inch LED monitor with a resolution of 1,680 x 1050p and a refresh rate of 60 Hz, running on a Windows PC and equipped with a keyboard and a mouse. Participants were seated approximately 25 inches from the computer screen. The IUI was developed as a dynamic Web application with AngularJS and was presented on Google Chrome in full-screen mode. A rule engine was implemented in the Web application to enable switching between the experimental (adaptive IUI) and control (regular IUI) conditions. Adaptive rules (see *Section 3.2.5*) were stored in a JSON file and loaded automatically upon selection of the experimental condition. When either condition was selected, a unique link was created for each participant that led to the appropriate interface version, and placeholder database entries were created to store the data. This data could be extracted directly from the IUI as a JSON file for subsequent analysis.

#### 3.3.2 The passive BCI

The BCI model was created using Simulink and MATLAB (version R2021b, Mathworks, MA) with the g.HIsys environment (g.tec medical engineering GmbH, Austria), which enables high-speed online data processing. The BCI system ran on a Windows PC operated by the researchers. Upon opening the BCI model, a folder was created for each participant number to store EEG data. The *n*-back task was integrated and directly accessible from the Simulink model. Cognitive load thresholds derived from the *n*-back are stored in the participant’s folder after task completion for integration into the BCI model.

**Figure 6:**
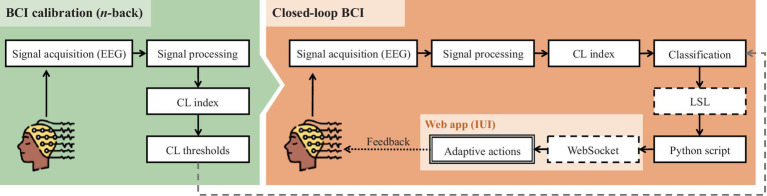
Visual representation of the BCI system operation, with the calibration task (n-back).

The BCI system operated as a closed-loop mode, continuously measuring cognitive load and adapting the speed of information presentation on the IUI (Figure 6). The BCI acquires and processes EEG signal, extracting features of alpha and theta band activity from the P7 electrode. The extracted features are then used to compute the cognitive load index. These stabilized values are compared to dynamic thresholds, resulting in a classification of three potential levels: 0, 1, and 2. The classifications are then transmitted every 6 s using Lab Streaming Layer (LSL) to a Python script. The script transmits the classifications to the interface every six seconds through WebSocket communication. Subsequently, the classifications are utilized in a rule engine to trigger the appropriate adaptive actions.

While only activity within the alpha and theta bands of the P7 electrode was considered in the analysis and classification of cognitive load, the BCI system monitored and stored brain activity from all 32 electrodes. The BCI stores the filtered P7 signals and the raw EEG data separately, which can be retrieved for post-hoc analyses. Finally, the BCI enables real-time visualization of EEG signals during the calibration and learning tasks to monitor signal quality and potential artifacts.

#### 3.3.3 EEG real-time processing

**Figure 7:**
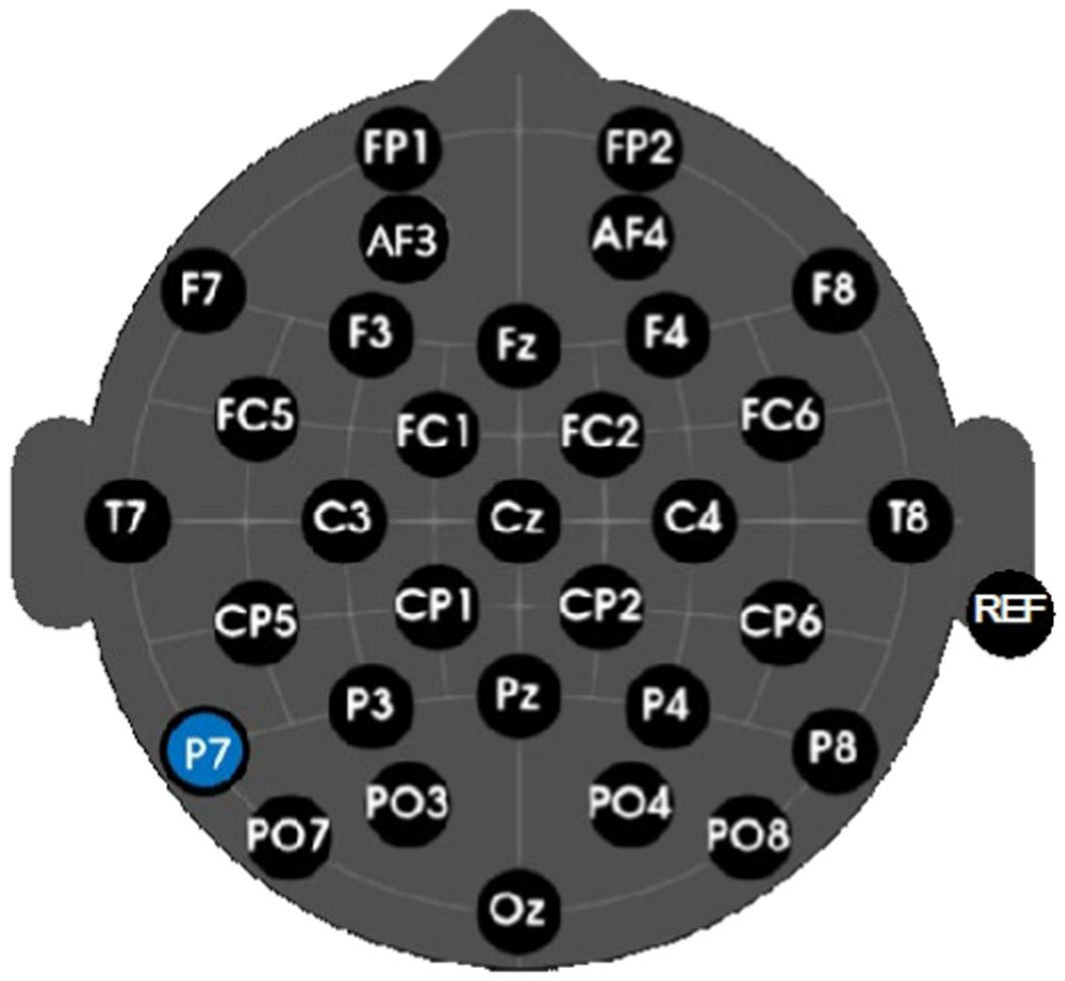
Electrode positioning of the EEG cap, P7 Indicated in blue and reference electrode denoted as REF.

Brain activity was continuously sampled using an active, 32-channel wireless and gel-based g.Nautilus Research EEG headset (g.tec medical engineering GmbH, Austria) with g.Scarabeo electrodes (Standard 10–20 System placement, see Figure 7). The EEG amplifier was secured in a holder shell at the base of the cap and fixed with Velcro. The real-time sampling rate was set to 250 Hz and filtered using bandpass (0.5 Hz – 30 Hz) and notch (58 Hz – 62 Hz) filters applied in real-time. Each electrode was equipped with an amplifier to enhance signal quality, minimize artifacts, and reduce signal degradation. The reference electrode was placed on the participant’s right earlobe to aid in common-mode rejection.

### 3.4 Psychometric instruments

Questionnaires were administered to the participants using Qualtrics (Qualtrics, Provo, UT) via anonymous links. Prior to completion, participants were required to enter their participant number, for later anonymous identification and analysis. Table 2 presents a summary of the questionnaires used in this study, including the degree of internal consistency for questionnaires with multiple items, which was assessed via Cronbach’s alpha (*α*).

**Table 2:** Questionnaires used in this study, with Cronbach’s alpha measure for multiple-item questionnaires.

Measure	Questionnaires used	Items	Cronbach’s alpha
Before the experiment			
Student status	Yes or no question	1	–
Prior level of interest	10-point Likert scale	1	–
Knowledge on constellations	Short, reliable measure of subjective Knowledge questionnaire	10	0.772
After the experiment			
Self-perceived usability of interface	System usability scale	10	0.798
Self-perceived mental workload	NASA-TLX	6	0.715
Self-perceived cognitive absorption	Cognitive absorption questionnaire	15	0.840
Self-perceived satisfaction	5-point Likert scale	1	–

#### 3.4.1 Pre-test questionnaire

The pre-test questionnaire collected demographic information and assessed participants’ prior knowledge and interest level in the learning topic. First, the questionnaire requested participants to enter their age (in years) and indicate the gender with which they identify to. Then, a simple Yes or No question evaluated the learner status (whether the participant is a student). Answers were converted into binary data, where 0 represented No, and 1 represented Yes. The prior level of interest in the learning topic was assessed with a 10-point Likert scale ranging from 1 “No interest” to 10 “Very interested.” Finally, knowledge of learning topic was assessed using a 10-item questionnaire adapted to the learning topic using a Likert scale ranging from 1 “Strongly disagree” to 7 “Strongly agree” (Flynn and Goldsmith, 1999). All items were averaged to create individual overall scores, where the higher the scores, the higher the prior level of knowledge. The internal consistency analysis revealed a questionable Cronbach’s alpha value (*α* = 0.709). Therefore, the second item was discarded to increase the internal consistency to a higher, more acceptable level (*α* = 0.772).

#### 3.4.2 Post-test questionnaire

Perceived mental workload was evaluated after the experiment with the raw NASA-TLX questionnaire (Hart and Staveland, 1988), composed of six dimensions: mental demand, physical demand, temporal demand, performance, effort, and frustration. A single item represented each dimension. Participants were asked to complete each item based on their learning experience. All dimensions were measured using slidable cursors on a continuous scale ranging from 0 to 100. Scores were rounded in post-hoc analyses to fit the questionnaire’s original calculations (Hart, 1986). This allowed for an overall mental workload score to be obtained, as well as individual observations of each dimension (Galy et al., 2018). The initial Cronbach’s alpha (*α* = 0.689) calculation showed moderate internal consistency. We removed the physical demand dimension to achieve an acceptable alpha of (*α* = 0.715). Removing an item is acceptable when using the raw NASA-TLX (Colligan et al., 2015; Hart, 2006).

Perceived cognitive absorption was measured after the experiment using an adapted version of the Cognitive Absorption questionnaire (Barki et al., 2008). This questionnaire covers the five dimensions, temporal dissociation, focused immersion, heightened enjoyment, control, and curiosity, as described by Agarwal and Karahanna (2000), to assess cognitive absorption, with three items per dimension. Items were measured with 7-point Likert scales, ranging from 1 “Strongly Disagree” to 7 “Strongly Agree.” An overall average score and an average score of each dimension were calculated and interpreted with high internal consistency Cronbach’s alpha (*α* = 0.840).

Perceived satisfaction was measured with a simple 5-point Likert scale ranging from 1- “not at all satisfied” to 5 “very satisfied” adapted from the Customer Satisfaction Score (CSAT) (Kiradoo, 2019).

Finally, subjective usability, as the user’s perception of how simple and effective it is to use the learning interface (Vlachogianni and Tselios, 2022), was measured using the System Usability Scale (SUS) (Brooke, 1996), with ten items over three dimensions: effectiveness, efficiency, and satisfaction (ISO 9241-11). All items were evaluated on a 1–5 Likert scale ranging from 1 “Strongly disagree” to 5 “Strongly agree.” Scores were then converted to scores ranging from 0–100 using the original calculation (Brooke, 1996). The internal consistency tests revealed an acceptable Cronbach’s alpha (*α* = 0.798).

#### 3.4.3 Learning gains

The participants’ answers to all questions for the learning task were extracted after task completion to measure the evolution of the learning gains throughout the experiment. A score of 1 or 0 was assigned for each correct or incorrect answer, respectively. All scores were compiled into a single file, and participants were associated with their performance data per block. Finally, learning gains for each participant were calculated by subtracting the scores of block 1 from the scores of block 2, block 3, and block 4.

### 3.5 Procedure

The average experimental session lasted approximately two hours. Participants were first greeted, provided with an explanation of the study, and then signed consent to participate. The experiment took place in a custom-built soundproof Faraday cage to protect the EEG signal from external electromagnetic interference. The experiment was monitored through a one-way mirror and shared computer screens in an adjacent room. Participants were seated in a chair in front of a computer screen, and a keyboard and mouse were provided to interact with the IUI. Once seated, participants were asked to complete the pre-test questionnaire. Shortly after, their head measurements were taken to fit the EEG cap and sensors, the amplifier was turned on, and electroconductive gel was applied to each electrode before impedance testing (< 7kOhm). The BCI model was then started, and the participants’ file was created to save their EEG data. Consequently, the researcher selected the correct interface type (regular or adaptive) in the IUI according to the participant’s number, which created the participant’s file in the learning interface.

Participants began with a calibration phase, consisting of the 90-s baseline task, followed by the *n*-back task to personalize cognitive load thresholds. The calibration phase was directly followed by the learning task, where participants first had to read the study instructions on the IUI’s landing page and wait for the researchers’ signal to start the task. They were asked to sit in a comfortable position and to limit head and body movements. For the AM group, participants were informed that their overall performance would be evaluated and that they should aim for the highest score possible to gain more prize draw tickets. Participants then started the task, which consisted of 4 blocks separated by 30-s breaks. After the experiment, participants were asked to complete a post-test questionnaire.

## 4 Data analysis

All statistical analyses were performed using R Studio (version 1.4.1103) using the *jamovi* package (version 1.2.23) to produce descriptive statics for cognitive load values of the learning task (derived from blocks 2 and 3), psychometric values and learning gains. The *psych* package (version 2.0.12) was used to calculate Cronbach’s alpha for the multiple-item questionnaires.

Initial data assessment showed that the data were ordinal, we thus opted for non-parametric statistical tests. We employed one-way independent samples Mann–Whitney U tests to compare adaptive measures, post-test questionnaire scores and learning gains between each group, using the *wilcox.test* function of the *stats* package (version 3.6.3). For single-tailed hypothesis, all *p* values obtained were divided by 2. For two-tailed hypotheses such as those involving level of interest and knowledge (see *Section 3.4.1*), measures of adaptivity and the measures derived from the SUS post-test questionnaire (see *Section 3.4.2*) *p*-values were not divided by 2. Finally, all effect sizes were calculated using the *wilcox_effsize* of the *rstatix* package (version 0.7.2), which returns the rank-biserial correlation by calculating *r* = z/√N (Rosenthal et al., 1994).

## 5 Results

### 5.1 Descriptive results of adaptive measures

We performed a Mann–Whitney U test to validate the effectiveness of the neuro-adaptive interface and assess whether adaptive measures occurred in response to changes in cognitive load. Table 3 provides a summary of the results for the adaptive measures under both adaptive conditions.

**Table 3:** Descriptive analysis of adaptive measures for both adaptive groups across blocks 2 and 3.

	Group A (*n* = 22)					Group AM (*n* = 16)					Mann–Whitney U	
	M	SD	Mdn	Max	Min	M	SD	Mdn	Max	Min	*U*	*p*
Block 2												
Total	33.55	8.77	34.00	52.00	16.00	32.94	7.18	33.00	47.00	21.00	179.50	0.929
Low CL	15.73	4.72	15.50	26.00	7.00	15.38	3.70	15.00	23.00	9.00	179.00	0.941
High CL	17.83	4.10	18.50	26.00	9.00	17.56	3.52	18.00	24.00	12.00	187.00	0.755
Block 3												
Total	34.18	8.46	34.50	48.00	19.00	33.88	6.93	35.00	47.00	23.00	177.50	0.976
Low CL	15.82	4.25	16.00	23.00	8.00	15.81	3.37	16.00	22.00	11.00	175.00	0.988
High CL	18.36	4.24	18.50	25.00	11.00	18.06	3.62	19.00	25.00	12.00	181.50	0.881
Blocks 2 and 3 combined												
Total	67.73	15.07	66.50	100.00	45.00	66.81	9.08	69.00	81.00	50.00	169.00	0.848
Low CL	31.55	7.94	31.00	49.00	20.00	31.19	4.55	32.00	38.00	22.00	162.50	0.700
High CL	36.18	7.20	36.00	51.00	24.00	35.63	4.63	37.00	43.00	26.00	175.00	0.988

The performance of the neuroadaptive interface across both experimental groups revealed no significant difference in its effectiveness (*p* > 0.05), suggesting its consistent responsiveness regardless of the presence or absence of the motivational factor. Overall, these results confirm that the IUI functioned as intended by adapting the speed of information provision for approximately half of the 64 constellations presented throughout each block when high and low cognitive load levels were detected, with comparable frequency on average for both low and high cognitive load levels across conditions.

### 5.2 Prior levels of interest and knowledge of constellations and perceived usability of the IUI

We performed further statistical testing to verify no differences exist between the three groups for the independent control pretest variables, prior level of interest, knowledge of constellations and perceived usability.

For prior level of interest, no significant differences were reported between the three groups (*p* > 0.05), group C (Mdn = 5.00), group A (Mdn = 5.00) and group AM (Mdn = 4.50). Similarly, for knowledge of constellations no significant differences were reported between the three groups (*p* > 0.05), group C (Mdn = 3.22), group A (Mdn = 3.00) and group AM (Mdn = 3.33).

Furthermore, no significant differences between the three groups were reported for the perceived usability of the interface (*p* > 0.05), group C (Mdn = 80.00), group A (Mdn = 78.75) and group AM (Mdn = 85.00). Moreover, the usability scores indicated above the “good usability” threshold of the scale’s interpretation (Brooke, 1996), group C (M = 82.06, SD = 8.02), group A (M = 77.95, SD = 10.98) and group AM (M = 81.09, SD = 13.51), confirming that the perceived usability of the interface did not influence.

### 5.3 Learning gains

**Figure 8:**
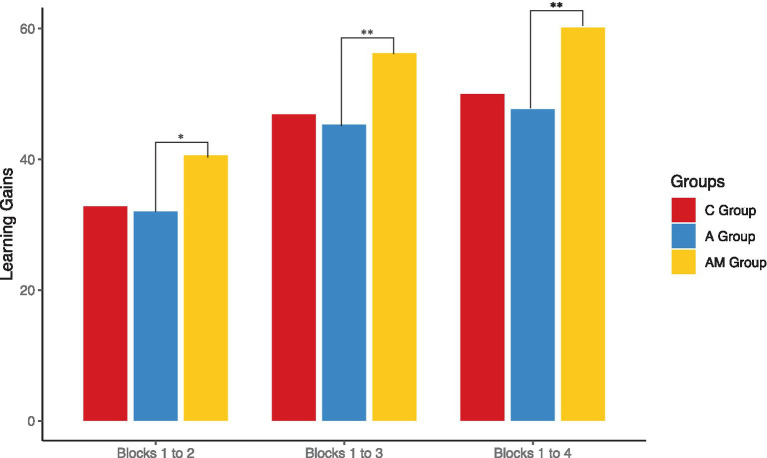
Learning gains throughout the experiment for each group (* *p* < 0.05, ** *p* < 0.01).

As indicated in Figure 8, group C and AM achieve greater learning gains than group A. Specifically, the AM group achieved the greatest learning gains. Statistical testing of the learning gains between groups C and A revealed no significant difference (*p* > 0.05) (Table 4), providing no support for our hypothesis (H1a), which states that neuro-adaptivity leads to greater learning gains compared to the absence of neuro-adaptivity. However, the AM group had significantly higher learning gains than group A between block 1 and 2 (*U* = 109.50, *p* = 0.023, *r* = 0.32), between block 1 and 3 (*U* = 88.00, *p* = 0.005, *r* = 0.42), and between block 1 and 4 (*U* = 70.00, *p* = 0.001, *r* = 0.51). Indicating that as the learning task progressed, the effect size became stronger, suggesting a greater impact of the motivational factor on the adaptive intervention. These findings support our hypothesis (H2a), which states that adding motivation to neuro-adaptivity helps to achieve greater learning gains compared to neuro-adaptivity alone.

**Table 4:** Descriptive statistics and between-subjects analyses for the learning gains (* *p* < 0.05, ** *p* < 0.01).

	Research question 1: comparing group C and group A (*n* = 39)								
	Group C (*n* = 17)			Group A (*n* = 22)			Mann–Whitney U test		
	M	SD	Mdn	M	SD	Mdn	*U*	*p*	*r*
Between Block 1 and 2	34.93	10.30	32.81	33.24	12.41	32.03	200.00	0.362	-
Between Block 1 and 3	46.78	11.27	46.88	45.88	11.60	45.31	194.50	0.423	-
Between Block 1 and 4	51.01	8.68	51.01	48.79	10.32	47.66	209.00	0.273	-

	Research question 2: comparing group A and group AM (*n* = 38)								
	Group A (*n* = 22)			Group AM (*n* = 16)			Mann–Whitney U test		
	M	SD	Mdn	M	SD	Mdn	*U*	*p*	*r*
Between Block 1 and 2	33.24	12.41	32.03	40.72	9.45	40.63	109.50	0.023 *	0.32
Between Block 1 and 3	45.88	11.60	45.31	55.76	9.42	56.25	88.00	0.005 **	0.42
Between Block 1 and 4	48.79	10.32	47.66	60.45	8.85	60.16	70.00	0.001 **	0.51

Learning gains range from 0–100.

### 5.4 Perceived mental workload

Based on the perceived cognitive workload questionnaire’s interpretation table (Hart, 1986), groups A and AM reported a “somewhat high” mean score of perceived mental workload, while group C reported a “somewhat high” to “borderline high” mean score (Table 5), indicating the highest level of mental workload. However, no significant difference between groups C and A, nor between groups A and AM for the overall questionnaire (*p* > 0.05) were reported. These findings provide no support for our hypothesis (H2b), which states that adding motivation to neuro-adaptivity reduces perceived mental workload compared to neuro-adaptivity alone. However, individual analysis for the Temporal Demand dimension reported a significant difference between group A and C (*U* = 268.50, *p* = 0.012, *r* = 0.37) in that, participants in group A reported feeling significantly less time pressure. These findings partially support our hypothesis (H1b), which states that neuro-adaptivity reduces perceived mental workload compared to the absence of neuro-adaptivity.

**Table 5:** Descriptive statistics and between-subjects analyses for the perceived mental workload (* *p* < 0.05, ** *p* < 0.01).

	Research question 1: comparing group C and group A (*n* = 39)								
	Group C (*n* = 17)			Group A (*n* = 22)			Mann–Whitney U test		
	M	SD	Mdn	M	SD	Mdn	*U*	*p*	*r*
Perceived mental workload (0–100)	48.29	14.42	50.00	40.05	13.54	39.50	244.00	0.055	-
Mental demand	56.76	20.84	60.00	46.82	23.12	45.00	234.00	0.093	-
Temporal demand	53.53	19.26	55.00	38.18	24.71	30.00	268.50	0.012 *	0.37
Performance	30.29	17.54	25.00	27.05	16.95	25.00	211.50	0.247	-
Effort	62.35	22.92	65.00	60.45	18.32	67.50	205.00	0.309	-
Frustration	38.53	23.17	40.00	27.73	23.49	20.00	242.00	0.061	-

	Research question 2: comparing group A and group AM (*n* = 38)								
	Group A (*n* = 22)			Group AM (*n* = 16)			Mann–Whitney U test		
	M	SD	Mdn	M	SD	Mdn	*U*	*p*	*r*
Perceived mental workload (0–100)	40.05	13.54	39.50	41.06	16.81	41.00	179.00	0.471	-
Mental demand	46.82	23.12	45.00	44.38	28.22	30.00	192.00	0.323	-
Temporal demand	38.18	24.71	30.00	44.69	26.92	40.00	152.00	0.242	-
Performance	27.05	16.95	25.00	26.56	15.68	25.00	172.50	0.465	-
Effort	60.45	18.32	67.50	55.63	21.67	60.00	205.00	0.199	-
Frustration	27.73	23.49	20.00	34.06	22.38	30.00	141.50	0.155	-

### 5.5 Perceived cognitive absorption

No significant difference between groups C, A and AM were reported for overall perceived cognitive absorption (*p* > 0.05) (Table 6). However, individual analysis of the Heightened Enjoyment dimension reported that group A reported feeling significantly less enjoyment in completing the learning task than group C (*U* = 292.5, *p* = 0.002, *r* = 0.48). These findings do not support our hypothesis (H1c), which states that neuro-adaptivity generates a higher level of self-perceived cognitive absorption compared to the absence of neuro-adaptivity. For the same dimension, the AM group reported a significantly higher level of enjoyment compared to the A group (*U* = 118.00, *p* = 0.044, *r* = 0.28). Additionally, individual analysis of the Curiosity dimension revealed that the AM group reported feeling significantly more curious about constellations and the learning interface compared to the A group (*U* = 97.50, *p* = 0.011, *r* = 0.38), partially supporting our hypothesis (H2c), which states that adding motivation to neuro-adaptivity generates a higher level of self-perceived cognitive absorption than neuro-adaptivity alone.

**Table 6:** Descriptive statistics and between-subjects analyses for the perceived cognitive absorption (* *p* < 0.05, ** *p* < 0.01).

	Research question 1: comparing group C and group A (*n* = 39)								
	Group C (*n* = 17)			Group A (*n* = 22)			Mann–Whitney U test		
	M	SD	Mdn	M	SD	Mdn	*U*	*p*	*r*
Perceived cognitive absorption (1–7)	4.67	0.76	4.47	4.27	0.85	4.23	245.00	0.052	–
Temporal dissociation	3.98	1.31	4.00	3.52	1.26	3.17	230.50	0.111	–
Focused immersion	5.12	1.25	5.00	4.94	1.53	5.17	190.00	0.472	–
Heightened Enjoyment	4.71	1.03	4.33	3.64	1.19	3.67	292.50	0.002 **	0.48
Curiosity	4.12	1.29	4.33	3.88	1.37	3.83	210.00	0.261	–
Control	5.43	0.89	5.67	5.36	0.72	5.33	207.50	0.284	-

	Research question 2: comparing group A and group AM (*n* = 38)								
	Group A (*n* = 22)			Group AM (*n* = 16)			Mann–Whitney U test		
	M	SD	Mdn	M	SD	Mdn	*U*	*p*	*r*
Perceived cognitive absorption (1–7)	4.27	0.85	4.23	4.70	0.72	4.67	120.50	0.052	–
Temporal dissociation	3.52	1.26	3.17	3.21	1.34	2.67	200.50	0.238	–
Focused immersion	4.94	1.53	5.17	5.60	0.60	5.67	138.00	0.133	–
Heightened enjoyment	3.64	1.19	3.67	4.35	1.43	4.67	118.00	0.044 *	0.28
Curiosity	3.88	1.37	3.83	4.81	1.10	5.00	97.50	0.011 *	0.38
Control	5.36	0.72	5.33	5.50	0.73	5.50	143.00	0.165	–

### 5.6 Perceived satisfaction

Groups C and AM reported a higher mean score of self-perceived satisfaction than group A (Table 7). Group C reported the highest mean score of the three groups. Statistical testing revealed that group A reported feeling significantly less satisfied with their learning experience compared to group C (U = 261.5, *p* = 0.014, r = 0.35). However, no significant difference was found between groups A and AM (*p* > 0.05). These findings do not support our hypothesis (H1d), which states that neuro-adaptivity generates a higher level of self-perceived satisfaction of the learning experience compared to the absence of neuro-adaptivity. Furthermore. these findings provide no support for our hypothesis (H2d), which states that adding motivation to neuro-adaptivity generates a higher level of self-perceived satisfaction of the learning experience compared to neuro-adaptivity alone.

**Table 7:** Descriptive statistics and between-subjects analyses for the perceived satisfaction (* *p* < 0.05, ** *p* < 0.01).

	Research question 1: comparing group C and group A (*n* = 39)								
	Group C (*n* = 17)			Group A (*n* = 22)			Mann–Whitney U test		
	M	SD	Mdn	M	SD	Mdn	*U*	*p*	*r*
Perceived satisfaction (1–5)	4.12	0.78	4.00	3.36	1.14	3.00	261.50	0.014 *	0.35

	Research question 2: Comparing group A and group AM (*n* = 38)								
	Group A (*n* = 22)			Group AM (*n* = 16)			Mann–Whitney U test		
	M	SD	Mdn	M	SD	Mdn	*U*	*p*	*r*
Perceived satisfaction (1–5)	3.36	1.14	3.00	3.81	1.05	4.00	132.50	0.093	–

## 6 Discussion

Our results suggest that adapting the learning speed of a memorization-based learning task, when combined with a motivational factor, leads to greater learning gains and greater curiosity and enjoyment when performing the learning task. It appears that motivation plays a role in influencing these results, and it is evident that it had a significant impact on the neuro-adaptive interface’s effectiveness. These results emphasize the importance of considering motivational strategies and interface design in developing adaptive learning interfaces to optimize learning experiences.

First, our results suggest that motivation plays a critical role in achieving greater learning gains. Even though participants used the same adaptive IUI, the AM group outperformed the A group. This finding could be explained by the presence of the motivating factor, which may have led participants to become more invested and persistent in completing the learning task. Furthermore, this result aligns with the current literature, suggesting that extrinsic motivation is important in improving test results (Liu et al., 2012). Extrinsic motivation has been suggested to cultivate motivation when beginning learning experiences, which may develop into intrinsic motivation as the learning process progresses (Tohidi and Jabbari, 2012). Potentially, participants in the AM group may have been motivated by the financial incentive at first, which may have grown into intrinsic motivation due to the length of the learning task. Therefore, in the current study, the financial incentive may have been a driving force to complete the learning task, which led to higher levels of enjoyment and curiosity as expected from intrinsic motivation. This conclusion is supported by research showing that while extrinsic motivational factors may not have as much of a long-term impact as intrinsic motivational factors, they can lead to high levels of engagement and commitment in the short term and better learning performances (Tohidi and Jabbari, 2012). Furthermore, a study by Robinson and colleagues investigated the impact of a financial incentive on attention and memory test performance; their results suggest that extrinsically motivated participants performed significantly better at both attention and memory tests (Robinson et al., 2012). These results support our findings that show a similar effect of greater learning gains from participants in the AM group in our memory-based learning task results.

However, contrary to our expectations, the adaptive IUI alone did not result in greater learning gains than the regular non-adaptive IUI. The results indicated that the adaptive IUI led to significantly lower enjoyment and overall satisfaction levels than the regular non-adaptive IUI. One possible explanation for these results is that the rapid learning pace (speed of information presentation) imposed on the control group may have served as an indirect extrinsic motivator. In other words, the quick information delivery speed may have been perceived as a competition, indirectly prompting and extrinsically motivating participants to race against the clock. Therefore, it is conceivable that those participants who used the adaptive IUI experienced a decrease in motivation, potentially due to the increased length of the task and repeated instructions, compared to those using the fast-imposed learning speed, who may have perceived and experienced the imposed rapid pace as an indirect extrinsic motivator. Thus, group A may have experienced increased boredom, negatively impacting the learning experience overall.

Comparatively, as the learning experiment progressed, it appears that regular IUI users became more accustomed to the swift delivery pace. However, the imposed pace did not lead to lower learning gains as expected; instead, it appears to have enhanced the enjoyment and satisfaction of the experience due to a possible indirect effect on the learner’s extrinsic motivation. This finding aligns with current literature, suggesting that a motivated learner may have higher satisfaction and pleasure levels while completing a task (Borah, 2021). Furthermore, the AM group, which coupled interface adaptivity with financial gain, may have overlooked the length of the task and the repetitive instructions due to increased immersion and the added extrinsic motivational factor, which gave a purpose to pursue and finish the learning task. Consequently, the AM group reported significantly higher enjoyment and curiosity, contributing to a greater learning experience.

Task difficulty may also have affected the classification of the cognitive load index and the relationship between alpha and theta activity, which may have had downstream effects on how responsive the adaptive interface was to changes in cognitive workload at the participant level. As mentioned in *Section 2.3*, alpha desynchronization is known to result from cognitive processing in situations of moderate to high mental workload during memory-based learning tasks. However, in the current study, some participants may have struggled with the task, leading to the solicitation of additional resources from the brain to cope with the heightened cognitive load. Past studies have shown that during more demanding cognitive tasks, theta synchronization may obscure alpha desynchronization in the context of cognitive load, leading to measurement issues (Klimesch, 1999; Klimesch et al., 1998). In other words, increased task difficulty enhances theta synchronization, resulting in the inhibition of alpha desynchronization within regions of the brain measured by EEG. In a word-memorization study by Klimesch et al. (1997), they found a connection between theta synchronization and the encoding and retrieval of episodic information. These findings point to a potential limitation in the design of the BCI used in this study, given that the classification index used only considers alpha activity at the parietal P7 electrode (see *Section 6.1*).

Furthermore, our findings partially support our hypothesis that employing the adaptive IUI leads to a decreased mental workload compared to the regular non-adaptive IUI. Even though no significant differences were observed in the global score of the mental workload questionnaire between groups A and C, the Temporal Demand dimension did indicate a greater level of temporal stress in group C. In other words, group C felt significantly more time-restricted and felt hurried and rushed to complete the learning task. More precisely, group C may have found the learning task more challenging as they needed to manage their cognitive load resources while keeping up with the fast-imposed pace of the learning task. This result aligns with the working memory resource depletion hypothesis, which suggests that learning tasks requiring active use of working memory resources may lead to temporary depletion and fatigue and can place additional stress on the learner (Chen et al., 2018). Overall, our results demonstrate the effectiveness of adjusting the speed of information presentation, i.e., learning pace, to the learner’s real-time cognitive load to reduce the perception of temporal stress of the user.

### 6.1 Limitations and future work

First, our cognitive load classification index only includes alpha-band activity at the parietal P7 electrode. This decision was made based on the analysis of our pre-tests and confirmed by the current literature (see *Section 3.2.4*). However, we acknowledge that this classification approach has limitations since cognitive load induces changes in brain activity within and between multiple cerebral regions, and our memory-based learning task demands not only information encoding and retrieval but also rapid decision-making as participants must identify the correct constellation name when a constellation image is presented. Decision-making requires manipulating multiple pieces of information to make a decision, significantly impacting working memory capacity. Previous studies indicate that the prefrontal cortex plays a central role in decision-making processes, specifically with alpha and theta oscillations (Bechara et al., 1998; Euston et al., 2012). Therefore, in future work, we shall analyze the functional connectivity between parietal and prefrontal activity to measure both cognitive load and decision-making processes in real-time, revealing how information is processed and integrated. Changes in connectivity indicate adjustments in cognitive load during memory-based learning tasks (Katsuki and Constantinidis, 2012; Murray et al., 2017; Vincent et al., 2008).

Second, our BCI model did not include an EEG signal artifact filtering block. To minimize the occurrence of artifacts, we monitored electrode impedances and the EEG signal constantly during the session. Additionally, we limited external inferences by conducting the experiment within a Faraday cage. We used active EEG electrodes, including amplifiers, to minimize artifacts and signal degradation. We referred our signal to an electrode placed on the earlobe for common rejection mode. We integrated a data pre-processing block into the BCI model that had filters targeting specific relevant frequency bands. Finally, we instructed the participant to minimize body movements to ensure the validity of our results. Furthermore, our index has the advantage of stabilizing cognitive load classification by considering the last 60 s of recording, thus reducing artifact impact on the classification. Nevertheless, we acknowledge that the EEG signal quality used in the experiment might have been affected occasionally by some artifacts or muscle noise.

Finally, our study’s experimental design did not include a fourth group specifically tailored to investigate the impact of motivation in the absence of adaptive measures. The decision to include only three groups in our design was influenced by practical considerations, such as resource availability, and by existing theoretical foundations. Previous studies conducted in learning contexts without BCI technology have demonstrated that extrinsic and intrinsic motivation significantly impact learning gains and performance (Gong et al., 2021; Liu et al., 2012; Xu et al., 2021). Therefore, this design choice aimed to maintain a focused examination of the independent and interactive effects of adaptive measures and motivation on learning gains. In other words, the primary focus of this study was not the effect of motivation on learning with the regular IUI, as this has already been exhaustively studied and found to have a significant impact. However, we recognize that the inclusion of a fourth group of participants who complete the learning experience using the regular IUI and with the presence of the motivational factor could provide deeper insight into the interaction between the adaptive measures and motivation and could enhance the overall interpretation of the results.

In the future, improving the classification of cognitive load by considering brain networks instead of solely focusing on the alpha activity of the P7 electrode and integrating Machine Learning or Deep Learning tools into the BCI would be beneficial (Rabbani and Islam, 2024; Torres-García et al., 2023). For example, Gogna et al. (2024) used a Support Vector Machine (SVM) model to classify cognitive workload levels based on physiological data (EEG) and subjective assessments (NASA-TLX) during a “Spot the Difference” task. The SVM model demonstrated impressive classification accuracy, suggesting that it can effectively differentiate between varying levels of cognitive workload. Such a model could be tested when applied to a learning task. Additionally, integrating more advanced artifact cleaning methods into the online BCI model would be relevant to ensure thorough data cleaning (Barachant et al., 2013; Daly et al., 2014; Urigüen and Garcia-Zapirain, 2015). These improvements would lead to more efficient and granular cognitive load classification by considering different brain regions and frequency bands free from artifacts. Including a secondary physiological measure for classifying cognitive load or evaluating system performance, such as pupillometry data, would be valuable. This addition would yield a more comprehensive assessment of cognitive load and the impacts of the system on learning experiences and outcomes. In practice, it would also be interesting to evaluate this system among student populations with academic challenges, such as those with neurodevelopmental disorders like attention deficit disorder (with or without hyperactivity). Such a system could be a game-changer for learners who face academic challenges, as it would enable adaptive learning that caters to their abilities.

## 7 Conclusion

We designed this study to investigate the impact of a neuro-adaptive interface on the enhancement of the learning experience using a constellation memorization-based learning task. Our aim was to determine if a passive BCI, which adjusts the speed of presenting information to learners based on their real-time cognitive load levels, would enhance their learning experience by keeping them within their ZPD. Additionally, we explored to what extent motivation was a prerequisite for effective adaptation. Our study employed a between-subjects design. Participants were assigned to either the control group, adaptive without motivation group, or adaptive with motivation group based on their order of enrollment in the study. Before the experiment, all participants completed a pre-test questionnaire and the *n*-back task to calibrate personal cognitive load thresholds. These thresholds were subsequently utilized in only the two adaptive groups. In line with previous research, we hypothesized that neuroadaptivity creates an optimal learning environment by enhancing learning gains, reducing self-perceived cognitive workload, generating higher levels of self-perceived cognitive absorption, and generating a higher level of satisfaction about the learning experience. Finally, we expected that motivation moderates the effect of neuroadaptation by augmenting its effectiveness and self-perception of an optimal learning environment. To test these hypotheses, we conducted one-way, non-parametric between-group analyses. Our results suggest that coupling motivation and adaptive IUI enhances learning gains for a memory-based learning task and contributes to enhancing the overall learning experience. However, we found no significant impact of the adaptive IUI alone in enhancing the learning experience. Nevertheless, we discovered that the imposed learning pace induced a significant temporal stress perception but significantly decreased the satisfaction level of the BCI. Our results suggest the importance of considering motivational strategies and interface design in developing adaptive learning interfaces to optimize learning experiences.

By using motivation as a catalyst, our system makes it possible to significantly improve learning gains while respecting the individual abilities of each learner. In theory, our system addresses the problem of lack of individual consideration and personalization of learning according to each learner. To our knowledge, few studies have explored the use of passive BCI systems in educational settings. Our study contributes to advancing knowledge by establishing a foundation for the application of such a system in learning. In practice, our study demonstrates the potential and feasibility of utilizing both motivation and passive BCI to improve learning outcomes and enhance the overall learning experience. Overall, our findings support the pursuit of such an opportunity.

## Data Availability

The raw data supporting the conclusions of this article will be made available by the authors, without undue reservation.

## References

[ref1] Abiri R. Borhani S. Sellers E. W. Jiang Y. Zhao X. (2019). A comprehensive review of EEG-based brain–computer interface paradigms. J. Neural Eng. 16:011001. doi: 10.1088/1741-2552/aaf12e, PMID: 30523919

[ref2] Agarwal R. Karahanna E. (2000). Time flies when you're having fun: cognitive absorption and beliefs about information technology usage. MIS Q. 24, 665–694. doi: 10.2307/3250951

[ref3] Ahn M. Lee M. Choi J. Jun S. C. (2014). A review of brain-computer Interface games and an opinion survey from researchers, Developers and Users. Sensors 14, 14601–14633. doi: 10.3390/s140814601, PMID: 25116904 PMC4178978

[ref4] Alamri H. A. Watson S. Watson W. (2021). Learning technology models that support personalization within blended learning environments in higher education. TechTrends 65, 62–78. doi: 10.1007/s11528-020-00530-3

[ref5] Anderson B. A. (2016). The attention habit: how reward learning shapes attentional selection. Ann. N. Y. Acad. Sci. 1369, 24–39. doi: 10.1111/nyas.12957, PMID: 26595376

[ref6] Anderson E. W. Potter K. C. Matzen L. E. Shepherd J. F. Preston G. A. Silva C. T. (2011). A user study of visualization effectiveness using EEG and cognitive load. Computer graphics forum, vol. 30. Oxford, UK: Blackwell Publishing Ltd., 791–800.

[ref7] Andreessen L. M. Gerjets P. Meurers D. Zander T. O. (2021). Toward neuroadaptive support technologies for improving digital reading: a passive BCI-based assessment of mental workload imposed by text difficulty and presentation speed during reading. User Model. User-Adap. Inter. 31, 75–104. doi: 10.1007/s11257-020-09273-5

[ref8] Angrisani L. Arpaia P. Esposito A. Gargiulo L. Natalizio A. Mastrati G.. (2021). Passive and active brain-computer interfaces for rehabilitation in health 4.0. Measurement 18:100246. doi: 10.1016/j.measen.2021.100246

[ref9] Anmarkrud Ø. Andresen A. Bråten I. (2019). Cognitive load and working memory in multimedia learning: conceptual and measurement issues. Educ. Psychol. 54, 61–83. doi: 10.1080/00461520.2018.1554484

[ref10] Antonenko P. Paas F. Grabner R. van Gog T. (2010). Using electroencephalography to measure cognitive load. Educ. Psychol. Rev. 22, 425–438. doi: 10.1007/s10648-010-9130-y

[ref11] Apicella A. Arpaia P. Frosolone M. Improta G. Moccaldi N. Pollastro A. (2022). EEG-based measurement system for monitoring student engagement in learning 4.0. Sci. Rep. 12:5857. doi: 10.1038/s41598-022-09578-y, PMID: 35393470 PMC8987513

[ref12] Aricò P. Borghini G. Di Flumeri G. Colosimo A. Bonelli S. Golfetti A.. (2016). Adaptive automation triggered by EEG-based mental workload index: a passive brain-computer interface application in realistic air traffic control environment. Front. Hum. Neurosci. 10:539. doi: 10.3389/fnhum.2016.00539 27833542 PMC5080530

[ref13] Ayres P. (2006). Using subjective measures to detect variations of intrinsic cognitive load within problems. Learn. Instr. 16, 389–400. doi: 10.1016/j.learninstruc.2006.09.001

[ref14] Barachant A. Andreev A. Congedo M. (2013). The Riemannian potato: An automatic and adaptive artifact detection method for online experiments using Riemannian geometry. TOBI workshop lV, (pp. 19–20).

[ref15] Barki H. Pare G. Sicotte C. (2008). Linking IT implementation and acceptance via the construct of psychological ownership of information technology. J. Inf. Technol. 23, 269–280. doi: 10.1057/jit.2008.12

[ref16] Bawa P. (2016). Retention in online courses: exploring issues and solutions—a literature review. SAGE Open 6:2158244015621777. doi: 10.1177/2158244015621777

[ref17] Bechara A. Damasio H. Tranel D. Anderson S. W. (1998). Dissociation of working memory from decision making within the human prefrontal cortex. J. Neurosci. 18, 428–437. doi: 10.1523/JNEUROSCI.18-01-00428.1998, PMID: 9412519 PMC6793407

[ref18] Beck J. E. (2005). Engagement tracing: using response times to model student disengagement. Artif. Intell. Educ. 125:88.

[ref19] Birbaumer N. Murguialday A. R. Weber C. Montoya P. (2009). Neurofeedback and brain–computer interface: clinical applications. Int. Rev. Neurobiol. 86, 107–117. doi: 10.1016/S0074-7742(09)86008-X 19607994

[ref20] Bonaci T. Calo R. Chizeck H. J. (2015). App Stores for the Brain: privacy and security in brain-computer interfaces. IEEE Technol. Soc. Mag. 34, 32–39. doi: 10.1109/MTS.2015.2425551

[ref21] Borah M. (2021). Motivation in learning. J. Crit. Rev. 8, 550–552.

[ref22] Brooke J. (1996). SUS-A quick and dirty usability scale. Usab. Eval. Indus. 189, 4–7.

[ref23] Brouwer A.-M. Hogervorst M. A. van Erp J. B. F. Heffelaar T. Zimmerman P. H. Oostenveld R. (2012). Estimating workload using EEG spectral power and ERPs in the n-back task. J. Neural Eng. 9:045008. doi: 10.1088/1741-2560/9/4/045008, PMID: 22832068

[ref24] Brunken R. Plass J. L. Leutner D. (2003). Direct measurement of cognitive load in multimedia learning. Educ. Psychol. 38, 53–61. doi: 10.1207/S15326985EP3801_7 12053529

[ref25] Butler A. C. Karpicke J. D. Roediger Iii H. L. (2008). Correcting a metacognitive error: feedback increases retention of low-confidence correct responses. J. Exp. Psychol. Learn. Mem. Cogn. 34, 918–928. doi: 10.1037/0278-7393.34.4.918, PMID: 18605878

[ref26] Buttfield A. Ferrez P. W. Millan J. R. (2006). Towards a robust BCI: error potentials and online learning. IEEE Trans. Neural Syst. Rehabil. Eng. 14, 164–168. doi: 10.1109/TNSRE.2006.875555, PMID: 16792284

[ref27] Caplan B. Mendoza J. E. (2011). “Edinburgh handedness inventory” in Encyclopedia of clinical neuropsychology. eds. Kreutzer J. S. DeLuca J. Caplan B. (New York: Springer), 928.

[ref28] Carabalona R. Grossi F. Tessadri A. Castiglioni P. Caracciolo A. de Munari I. (2012). Light on! Real world evaluation of a P300-based brain–computer interface (BCI) for environment control in a smart home. Ergonomics 55, 552–563. doi: 10.1080/00140139.2012.661083, PMID: 22455346

[ref29] Cavanagh J. F. Frank M. J. (2014). Frontal theta as a mechanism for cognitive control. Trends Cogn. Sci. 18, 414–421. doi: 10.1016/j.tics.2014.04.012, PMID: 24835663 PMC4112145

[ref30] Chaiklin S. (2003). The zone of proximal development in Vygotsky’s analysis of learning and instruction. Vygotsky’s Educ. Theor. Cult. Cont. 1, 39–64. doi: 10.1017/CBO9780511840975.004

[ref31] Chaudhary U. Birbaumer N. Ramos-Murguialday A. (2016). Brain–computer interfaces for communication and rehabilitation. Nat. Rev. Neurol. 12, 513–525. doi: 10.1038/nrneurol.2016.113 27539560

[ref32] Chen O. Castro-Alonso J. C. Paas F. Sweller J. (2018). Extending cognitive load theory to incorporate working memory resource depletion: evidence from the spacing effect. Educ. Psychol. Rev. 30, 483–501. doi: 10.1007/s10648-017-9426-2

[ref33] Chen J. Zhang D. Engel A. K. Gong Q. Maye A. (2017). Application of a single-flicker online SSVEP BCI for spatial navigation. PLoS One 12:e0178385. doi: 10.1371/journal.pone.0178385, PMID: 28562624 PMC5451069

[ref34] Chipchase L. Davidson M. Blackstock F. Bye R. Clothier P. Klupp N.. (2017). Conceptualising and measuring student disengagement in higher education: a synthesis of the literature. Int. J. High. Educ. 6, 31–42. doi: 10.5430/ijhe.v6n2p31

[ref35] Cohen M. X. (2017). Where does EEG come from and what does it mean? Trends Neurosci. 40, 208–218. doi: 10.1016/j.tins.2017.02.004, PMID: 28314445

[ref36] Colligan L. Potts H. W. Finn C. T. Sinkin R. A. (2015). Cognitive workload changes for nurses transitioning from a legacy system with paper documentation to a commercial electronic health record. Int. J. Med. Inform. 84, 469–476. doi: 10.1016/j.ijmedinf.2015.03.003, PMID: 25868807

[ref37] Csikszentmihalyi M. (1975). Beyond boredom and anxiety. San Francisco, CA, US: Jossey-Bass.

[ref38] Csikszentmihalyi M. (2014). “Toward a Psychology of Optimal Experience,” in Flow and the Foundations of Positive Psychology. ed. M. Csikszentmihalyi. (Dordrecht: Springer).

[ref39] Curum B. Khedo K. K. (2021). Cognitive load management in mobile learning systems: principles and theories. J. Comput. Educ. 8, 109–136. doi: 10.1007/s40692-020-00173-6

[ref40] Daly I. Scherer R. Billinger M. Müller-Putz G. (2014). FORCe: fully online and automated artifact removal for brain-computer interfacing. IEEE Trans. Neural Syst. Rehabil. Eng. 23, 725–736. doi: 10.1109/TNSRE.2014.2346621, PMID: 25134085

[ref41] Daly J. J. Wolpaw J. R. (2008). Brain–computer interfaces in neurological rehabilitation. Lancet Neurol. 7, 1032–1043. doi: 10.1016/S1474-4422(08)70223-0 18835541

[ref42] De Jong T. (2010). Cognitive load theory, educational research, and instructional design: some food for thought. Instr. Sci. 38, 105–134. doi: 10.1007/s11251-009-9110-0

[ref43] Debue N. van de Leemput C. (2014). What does germane load mean? An empirical contribution to the cognitive load theory. Front. Psychol. 5:1099. doi: 10.3389/fpsyg.2014.01099, PMID: 25324806 PMC4181236

[ref44] Dehais F. Ladouce S. Darmet L. Nong T.-V. Ferraro G. Torre Tresols J.. (2022). Dual passive reactive brain-computer Interface: a novel approach to human-machine Symbiosis. Front. Neuroergon. 3:824780. doi: 10.3389/fnrgo.2022.824780, PMID: 38235478 PMC10790872

[ref45] DeLeeuw K. E. Mayer R. E. (2008). A comparison of three measures of cognitive load: evidence for separable measures of intrinsic, extraneous, and germane load. J. Educ. Psychol. 100, 223–234. doi: 10.1037/0022-0663.100.1.223

[ref46] Demazure T. Karran A. Labonté-LeMoyne É. Léger P.-M. Sénécal S. Fredette M.. (2019). Sustained attention in a monitoring task: Towards a Neuroadaptative Enterprise system Interface. Cham: Information Systems and Neuroscience.

[ref47] Demazure T. Karran A. Léger P.-M. Labonté-LeMoyne É. Sénécal S. Fredette M.. (2021). Enhancing sustained attention. Bus. Inf. Syst. Eng. 63, 653–668. doi: 10.1007/s12599-021-00701-3

[ref48] Dimoka A. Davis F. D. Gupta A. Pavlou P. A. Banker R. D. Dennis A. R.. (2012). On the use of neurophysiological tools in IS research: developing a research agenda for NeuroIS. MIS Q. 36, 679–702. doi: 10.2307/41703475

[ref49] Dumford A. D. Miller A. L. (2018). Online learning in higher education: exploring advantages and disadvantages for engagement. J. Comput. High. Educ. 30, 452–465. doi: 10.1007/s12528-018-9179-z

[ref50] Eldenfria A. Al-Samarraie H. (2019). Towards an online continuous adaptation mechanism (OCAM) for enhanced engagement: an EEG study. Int. J. Hum. Comput. Interact. 35, 1960–1974. doi: 10.1080/10447318.2019.1595303

[ref51] El-Sabagh H. A. (2021). Adaptive e-learning environment based on learning styles and its impact on development students' engagement. Int. J. Educ. Technol. High. Educ. 18, 1–24. doi: 10.1186/s41239-021-00289-4

[ref52] Euston D. R. Gruber A. J. McNaughton B. L. (2012). The role of medial prefrontal cortex in memory and decision making. Neuron 76, 1057–1070. doi: 10.1016/j.neuron.2012.12.002, PMID: 23259943 PMC3562704

[ref53] Ferrer J. Ringer A. Saville K. Parris A. Kashi K. (2022). Students’ motivation and engagement in higher education: the importance of attitude to online learning. High. Educ. 83, 317–338. doi: 10.1007/s10734-020-00657-5

[ref54] Festinger D. S. Marlowe D. B. Croft J. R. Dugosh K. L. Arabia P. L. Benasutti K. M. (2009). Monetary incentives improve recall of research consent information: it pays to remember. Exp. Clin. Psychopharmacol. 17, 99–104. doi: 10.1037/a0015421, PMID: 19331486 PMC3218798

[ref55] Fini A. (2009). The technological dimension of a massive open online course: the case of the CCK08 course tools. Int. Rev. Res. Open Distrib. Learn. 10, 2–26. doi: 10.19173/irrodl.v10i5.643

[ref56] Flynn L. R. Goldsmith R. E. (1999). A short, reliable measure of subjective knowledge. J. Bus. Res. 46, 57–66. doi: 10.1016/S0148-2963(98)00057-5

[ref57] Galy E. Paxion J. Berthelon C. (2018). Measuring mental workload with the NASA-TLX needs to examine each dimension rather than relying on the global score: an example with driving. Ergonomics 61, 517–527. doi: 10.1080/00140139.2017.1369583, PMID: 28817353

[ref58] Gao X. Wang Y. Chen X. Gao S. (2021). Interface, interaction, and intelligence in generalized brain–computer interfaces. Trends Cogn. Sci. 25, 671–684. doi: 10.1016/j.tics.2021.04.003 34116918

[ref59] Gerjets P. Walter C. Rosenstiel W. Bogdan M. Zander T. O. (2014). Cognitive state monitoring and the design of adaptive instruction in digital environments: lessons learned from cognitive workload assessment using a passive brain-computer interface approach. Front. Neurosci. 8:385. doi: 10.3389/fnins.2014.00385, PMID: 25538544 PMC4260500

[ref60] Gevins A. Smith M. E. (2003). Neurophysiological measures of cognitive workload during human-computer interaction. Theor. Issues Ergon. Sci. 4, 113–131. doi: 10.1080/14639220210159717

[ref61] Ghafurian M. Reitter D. Ritter F. E. (2020). Countdown timer speed: a trade-off between delay duration perception and recall. ACM Transact. Comput. Hum. Interact. 27, 1–25. doi: 10.1145/3380961

[ref62] Gogna Y. Tiwari S. Singla R. (2024). Evaluating the performance of the cognitive workload model with subjective endorsement in addition to EEG. Med. Biol. Eng. Comput. 62, 2019–2036. doi: 10.1007/s11517-024-03049-4, PMID: 38433179

[ref63] Gong J. Liu T. X. Tang J. (2021). How monetary incentives improve outcomes in MOOCs: evidence from a field experiment. J. Econ. Behav. Organ. 190, 905–921. doi: 10.1016/j.jebo.2021.06.029

[ref64] Grimes D. Tan D. S. Hudson S. E. Shenoy P. Rao R. P. (2008). Feasibility and pragmatics of classifying working memory load with an electroencephalograph. Proceedings of the SIGCHI conference on human factors in computing systems, (pp. 835–844).

[ref65] Grizioti M. Kynigos C. (2020). “Computer-based learning, computational thinking, and constructionist approaches” in Encyclopedia of education and information technologies.ed. A. Tatnall (Switzerland AG: Springer), 355–371.

[ref66] Gu X. Cao Z. Jolfaei A. Xu P. Wu D. Jung T. P.. (2021). EEG-based brain-computer interfaces (BCIs): a survey of recent studies on signal sensing technologies and computational intelligence approaches and their applications. IEEE/ACM Trans. Comput. Biol. Bioinform. 18, 1645–1666. doi: 10.1109/TCBB.2021.3052811, PMID: 33465029

[ref67] Hancock P. A. Meshkati N. (1988). Human mental workload. Amsterdam North-Holland: Elsevier Science Publishers B.V.

[ref68] Hart S. G. (1986). NASA task load index (TLX). (NASA) Ames Research Center Moffett Field, CA United States.

[ref69] Hart S. G. (2006). NASA-task load index (NASA-TLX); 20 years later. Proceedings of the Human Factors and Ergonomics Society Annual Meeting, 50 (904). 904, 908

[ref70] Hart S. G. Staveland L. E. (1988). “Development of NASA-TLX (task load index): results of empirical and theoretical research” in Advances in psychology, vol. 52 eds. P. A. Hancock and N. Meshkati (Amsterdam North-Holland: Elsevier Science Publishers B.V.), 139–183.

[ref71] Hasler B. S. Kersten B. Sweller J. (2007). Learner control, cognitive load and instructional animation. Appl. Cogn. Psychol. 21, 713–729. doi: 10.1002/acp.1345

[ref72] Hedegaard M. (2012). “The zone of proximal development as basis for instruction” in An introduction to Vygotsky ed. H. Daniels (Cambridge, United Kingdom: Cambridge University Press), 234–258.

[ref73] Hogervorst M. A. Brouwer A. M. van Erp J. B. (2014). Combining and comparing EEG, peripheral physiology and eye-related measures for the assessment of mental workload. Front. Neurosci. 8:322. doi: 10.3389/fnins.2014.00322, PMID: 25352774 PMC4196537

[ref74] Hu A. Shewokis P. A. Ting K. Fung K. (2016). Motivation in computer-assisted instruction. Laryngoscope 126, S5–S13. doi: 10.1002/lary.26040 27307270

[ref75] Kalyuga S. Liu T.-C. (2015). Guest editorial: managing cognitive load in technology-based learning environments. J. Educ. Technol. Soc. 18, 1–8.

[ref76] Karran A. J. Demazure T. Leger P.-M. Labonte-LeMoyne E. Senecal S. Fredette M.. (2019). Toward a hybrid passive BCI for the modulation of sustained attention using EEG and fNIRS. Front. Hum. Neurosci. 13:393. doi: 10.3389/fnhum.2019.00393, PMID: 31780914 PMC6851201

[ref77] Katsuki F. Constantinidis C. (2012). Unique and shared roles of the posterior parietal and dorsolateral prefrontal cortex in cognitive functions. Front. Integr. Neurosci. 6:17. doi: 10.3389/fnint.2012.00017 22563310 PMC3342558

[ref78] Kerous B. Skola F. Liarokapis F. (2018). EEG-based BCI and video games: a progress report. Virtual Reality 22, 119–135. doi: 10.1007/s10055-017-0328-x

[ref79] Kiradoo G. (2019). Software engineering quality to enhance the customer satisfaction level of the organization. Int. J. Adv. Res. Eng. Technol. 10, 297–302.

[ref80] Klašnja-Milićević A. Vesin B. Ivanović M. Budimac Z. (2011). E-learning personalization based on hybrid recommendation strategy and learning style identification. Comput. Educ. 56, 885–899. doi: 10.1016/j.compedu.2010.11.001

[ref81] Klimesch W. (1999). EEG alpha and theta oscillations reflect cognitive and memory performance: a review and analysis. Brain Res. Rev. 29, 169–195. doi: 10.1016/S0165-0173(98)00056-3 10209231

[ref82] Klimesch W. Doppelmayr M. Russegger H. Pachinger T. Schwaiger J. (1998). Induced alpha band power changes in the human EEG and attention. Neurosci. Lett. 244, 73–76. doi: 10.1016/S0304-3940(98)00122-0 9572588

[ref83] Klimesch W. Doppelmayr M. Schimke H. Ripper B. (1997). Theta synchronization and alpha desynchronization in a memory task. Psychophysiology 34, 169–176. doi: 10.1111/j.1469-8986.1997.tb02128.x 9090266

[ref84] Klimesch W. Schack B. Sauseng P. (2005). The functional significance of theta and upper alpha oscillations. Exp. Psychol. 52, 99–108. doi: 10.1027/1618-3169.52.2.99, PMID: 15850157

[ref85] Kosmyna N. Maes P. (2019). AttentivU: an EEG-based closed-loop biofeedback system for real-time monitoring and improvement of engagement for personalized learning. Sensors 19:5200. doi: 10.3390/s19235200, PMID: 31783646 PMC6929136

[ref86] Kosmyna N. Tarpin-Bernard F. Bonnefond N. Rivet B. (2016). Feasibility of BCI control in a realistic smart home environment. Front. Hum. Neurosci. 10:416. doi: 10.3389/fnhum.2016.00416, PMID: 27616986 PMC4999433

[ref87] Krol L. R. Zander T. O. (2017). Passive BCI-based Neuroadaptive systems. Proceedings of the 7th Graz brain-computer Interface conference,

[ref88] Ku H.-Y. Sullivan H. J. (2002). Student performance and attitudes using personalized mathematics instruction. Educ. Technol. Res. Dev. 50, 21–34. doi: 10.1007/BF02504959

[ref89] Kulhavy R. W. (1977). Feedback in written instruction. Rev. Educ. Res. 47, 211–232. doi: 10.3102/00346543047002211

[ref90] Laar B. V. D. Gürkök H. Bos D. P.-O. Poel M. Nijholt A. (2013). Experiencing BCI control in a popular computer game. IEEE Transactions on Computational Intelligence and AI in Games, 5(2), 176–184.

[ref91] Labonte-Lemoyne E. Courtemanche F. Louis V. Fredette M. Sénécal S. Léger P.-M. (2018). Dynamic threshold selection for a biocybernetic loop in an adaptive video game context. Front. Hum. Neurosci. 12:282. doi: 10.3389/fnhum.2018.00282, PMID: 30065638 PMC6056683

[ref92] Lalor E. C. Kelly S. P. Finucane C. Burke R. Smith R. Reilly R. B.. (2005). Steady-state VEP-based brain-computer Interface control in an immersive 3D gaming environment. EURASIP J. Adv. Signal Proc. 2005:706906. doi: 10.1155/ASP.2005.3156

[ref93] Lécuyer A. Lotte F. Reilly R. B. Leeb R. Hirose M. Slater M. (2008). Brain-computer interfaces, virtual reality, and videogames. Computer 41, 66–72. doi: 10.1109/MC.2008.410

[ref94] Lepper M. R. Malone T. W. (2021). “Intrinsic motivation and instructional effectiveness in computer-based education,” in Aptitude, learning, and instruction. eds. R. E. Snow and M. J. Farr (London, United Kingdom: Routledge), 255–286.

[ref95] Liang S. F. Shaw F. Z. Young C. P. Chang D. W. Liao Y. C. (2010). A closed-loop brain computer interface for real-time seizure detection and control. 2010 annual international conference of the IEEE engineering in medicine and biology, (pp. 4950–4953). IEEE. 10.1109/IEMBS.2010.5627243 21096670

[ref96] Lin C. T. Lin B. S. Lin F. C. Chang C. J. (2014). Brain computer Interface-based smart living environmental auto-adjustment control system in UPnP home networking. IEEE Syst. J. 8, 363–370. doi: 10.1109/JSYST.2012.2192756

[ref97] Liu O. L. Bridgeman B. Adler R. M. (2012). Measuring learning outcomes in higher education: motivation matters. Educ. Res. 41, 352–362. doi: 10.3102/0013189X12459679

[ref98] Lotte F. Bougrain L. Cichocki A. Clerc M. Congedo M. Rakotomamonjy A.. (2018). A review of classification algorithms for EEG-based brain–computer interfaces: a 10 year update. J. Neural Eng. 15:31005. doi: 10.1088/1741-2552/aab2f2, PMID: 29488902

[ref99] Maksimenko V. A. Van Heukelum S. Makarov V. V. Kelderhuis J. Lüttjohann A. Koronovskii A. A.. (2017). Absence seizure control by a brain computer interface. Sci. Rep. 7:2487. doi: 10.1038/s41598-017-02626-y 28555070 PMC5447660

[ref100] Mamolo L. A. (2022). Online learning and students’ mathematics motivation, self-efficacy, and anxiety in the “new Normal”. Educ. Res. Int. 2022, 1–10. doi: 10.1155/2022/9439634

[ref101] Marchesi M. Riccò B. (2013). BRAVO: a brain virtual operator for education exploiting brain-computer interfaces. In CHI'13 extended abstracts on human factors in computing systems (pp. 3091–3094).

[ref102] Martin F. Bolliger D. U. (2022). Developing an online learner satisfaction framework in higher education through a systematic review of research. Int. J. Educ. Technol. High. Educ. 19, 1–21. doi: 10.1186/s41239-022-00355-535013716

[ref103] Mashrur F. R. Rahman K. M. Miya M. T. I. Vaidyanathan R. Anwar S. F. Sarker F.. (2022). BCI-based Consumers' choice prediction from EEG signals: an intelligent Neuromarketing framework. Front. Hum. Neurosci. 16:861270. doi: 10.3389/fnhum.2022.861270, PMID: 35693537 PMC9177951

[ref104] Mertens U. Finn B. Lindner M. A. (2022). Effects of computer-based feedback on lower-and higher-order learning outcomes: a network meta-analysis. J. Educ. Psychol. 114, 1743–1772. doi: 10.1037/edu0000764

[ref105] Murray J. D. Jaramillo J. Wang X.-J. (2017). Working memory and decision-making in a frontoparietal circuit model. J. Neurosci. 37, 12167–12186. doi: 10.1523/JNEUROSCI.0343-17.2017, PMID: 29114071 PMC5729190

[ref106] Mutlu-Bayraktar D. Cosgun V. Altan T. (2019). Cognitive load in multimedia learning environments: a systematic review. Comput. Educ. 141:103618. doi: 10.1016/j.compedu.2019.103618

[ref107] Najjar L. J. (1996). Multimedia information and learning. J. Educ. Multimed. Hypermedia 5, 129–150.

[ref108] Nikou S. A. Economides A. A. (2016). The impact of paper-based, computer-based and mobile-based self-assessment on students' science motivation and achievement. Comput. Hum. Behav. 55, 1241–1248. doi: 10.1016/j.chb.2015.09.025

[ref109] O'Byrne W. I. Pytash K. E. (2015). Hybrid and blended learning: modifying pedagogy across path, pace, time, and place. J. Adolesc. Adult. Lit. 59, 137–140. doi: 10.1002/jaal.463

[ref110] Paas F. Renkl A. Sweller J. (2004). Cognitive load theory: instructional implications of the interaction between information structures and cognitive architecture. Instr. Sci. 32, 1–8. doi: 10.1023/B:TRUC.0000021806.17516.d0

[ref111] Paas F. Tuovinen J. E. Van Merrienboer J. J. Aubteen Darabi A. (2005). A motivational perspective on the relation between mental effort and performance: optimizing learner involvement in instruction. Educ. Technol. Res. Dev. 53, 25–34. doi: 10.1007/BF02504795

[ref112] Padfield N. Zabalza J. Zhao H. Masero V. Ren J. (2019). EEG-based brain-computer interfaces using motor-imagery: techniques and challenges. Sensors 19:1423. doi: 10.3390/s19061423, PMID: 30909489 PMC6471241

[ref113] Papalambros N. A. Santostasi G. Malkani R. G. Braun R. Weintraub S. Paller K. A.. (2017). Acoustic enhancement of sleep slow oscillations and concomitant memory improvement in older adults. Front. Hum. Neurosci. 11:109. doi: 10.3389/fnhum.2017.00109 28337134 PMC5340797

[ref114] Pels E. G. M. Aarnoutse E. J. Leinders S. Freudenburg Z. V. Branco M. P. van der Vijgh B. H.. (2019). Stability of a chronic implanted brain-computer interface in late-stage amyotrophic lateral sclerosis. Clin. Neurophysiol. 130, 1798–1803. doi: 10.1016/j.clinph.2019.07.020, PMID: 31401488 PMC6880281

[ref115] Petko D. Schmid R. Cantieni A. (2020). Pacing in serious games: exploring the effects of presentation speed on cognitive load, engagement and learning gains. Simul. Gaming 51, 258–279. doi: 10.1177/1046878120902502

[ref116] Phan H. Andreotti F. Cooray N. Chén O. Y. Vos M. D. (2019). Joint classification and prediction CNN framework for automatic sleep stage classification. IEEE Trans. Biomed. Eng. 66, 1285–1296. doi: 10.1109/TBME.2018.2872652, PMID: 30346277 PMC6487915

[ref117] Pope A. T. Bogart E. H. Bartolome D. S. (1995). Biocybernetic system evaluates indices of operator engagement in automated task. Biol. Psychol. 40, 187–195. doi: 10.1016/0301-0511(95)05116-3, PMID: 7647180

[ref118] Rabbani M. H. R. Islam S. M. R. (2024). Deep learning networks based decision fusion model of EEG and fNIRS for classification of cognitive tasks. Cogn. Neurodyn. 18, 1489–1506. doi: 10.1007/s11571-023-09986-4, PMID: 39104699 PMC11297873

[ref119] Riedl R. Davis F. D. Hevner A. (2014). Towards a NeuroIS research methodology: intensifying the discussion on methods, tools, and measurement. J. Assoc. Inf. Syst. 15, I–XXXV. doi: 10.17705/1jais.00377

[ref120] Riedl R. Léger P.-M. (2016). “Tools in NeuroIS research: an overview” in Fundamentals of NeuroIS: Information systems and the brain. eds. R. Riedl and P. Léger (Berlin Heidelberg: Springer), 47–72.

[ref121] Riopel M. Chastenay P. Fortin-Clément G. Potvin P. Masson S. Charland P. (2017). Using invariance to model practice, forgetting, and spacing effects. Edulearn17 proceedings, (pp. 4334–4341). IATED.

[ref122] Robinson L. J. Stevens L. H. Threapleton C. J. Vainiute J. McAllister-Williams R. H. Gallagher P. (2012). Effects of intrinsic and extrinsic motivation on attention and memory. Acta Psychol. 141, 243–249. doi: 10.1016/j.actpsy.2012.05.012, PMID: 22738789

[ref123] Rosenthal R. Cooper H. Hedges L. (1994). Parametric measures of effect size. The handbook of research synthesis. eds. H. Cooper and L. Hedges (Russell Sage Foundation) 621, 231–244.

[ref124] Rosso O. A. Blanco S. Yordanova J. Kolev V. Figliola A. Schürmann M.. (2001). Wavelet entropy: a new tool for analysis of short duration brain electrical signals. J. Neurosci. Methods 105, 65–75. doi: 10.1016/S0165-0270(00)00356-3, PMID: 11166367

[ref125] Rousu M. C. Corrigan J. R. Harris D. Hayter J. K. Houser S. Lafrancois B. A.. (2015). Do monetary incentives matter in classroom experiments? Effects on course performance. J. Econ. Educ. 46, 341–349. doi: 10.1080/00220485.2015.1071214

[ref126] Roy R. N. Bonnet S. Charbonnier S. Campagne A. (2013). Mental fatigue and working memory load estimation: Interaction and implications for EEG-based passive BCI. 2013 35th annual international conference of the IEEE engineering in medicine and biology society (EMBC), (pp. 6607–6610). IEEE. 10.1109/EMBC.2013.6611070 24111257

[ref127] Ryan R. M. Deci E. L. (2000a). Intrinsic and extrinsic motivations: classic definitions and new directions. Contemp. Educ. Psychol. 25, 54–67. doi: 10.1006/ceps.1999.1020, PMID: 10620381

[ref128] Ryan R. M. Deci E. L. (2000b). Self-determination theory and the facilitation of intrinsic motivation, social development, and well-being. Am. Psychol. 55, 68–78. doi: 10.1037/0003-066X.55.1.68 11392867

[ref129] Saadé R. Bahli B. (2005). The impact of cognitive absorption on perceived usefulness and perceived ease of use in on-line learning: an extension of the technology acceptance model. Inf. Manag. 42, 317–327. doi: 10.1016/j.im.2003.12.013

[ref130] Salimon M. G. Sanuri S. M. M. Aliyu O. A. Perumal S. Yusr M. M. (2021). E-learning satisfaction and retention: a concurrent perspective of cognitive absorption, perceived social presence and technology acceptance model. J. Syst. Inf. Technol. 23, 109–129. doi: 10.1108/JSIT-02-2020-0029

[ref131] Schildberg-Hörisch H. Wagner V. (2020). Monetary and non-monetary incentives for educational attainment: design and effectiveness. Econ. Educ., 249–268. doi: 10.1016/B978-0-12-815391-8.00019-7

[ref132] Schnotz W. Kürschner C. (2007). A reconsideration of cognitive load theory. Educ. Psychol. Rev. 19, 469–508. doi: 10.1007/s10648-007-9053-4

[ref133] Schwab J. F. Somerville L. H. (2022). Raising the stakes for online learning: monetary incentives increase performance in a computer-based learning task under certain conditions. Front. Psychol. 13:301. doi: 10.3389/fpsyg.2022.780301, PMID: 35602677 PMC9119014

[ref134] Shabani K. Khatib M. Ebadi S. (2010). Vygotsky's zone of proximal development: instructional implications and teachers' professional development. Engl. Lang. Teach. 3, 237–248. doi: 10.5539/elt.v3n4p237

[ref135] Shemshack A. Spector J. M. (2020). A systematic literature review of personalized learning terms. Smart Learn. Environ. 7:33. doi: 10.1186/s40561-020-00140-9

[ref136] Small D. M. Gitelman D. Simmons K. Bloise S. M. Parrish T. Mesulam M.-M. (2005). Monetary incentives enhance processing in brain regions mediating top-down control of attention. Cereb. Cortex 15, 1855–1865. doi: 10.1093/cercor/bhi063, PMID: 15746002

[ref137] Spüler M. Rosenstiel W. Bogdan M. (2012). Online adaptation of a c-VEP brain-computer interface (BCI) based on error-related potentials and unsupervised learning. PLoS One 7:e51077. doi: 10.1371/journal.pone.0051077, PMID: 23236433 PMC3517594

[ref138] Steinert S. Bublitz C. Jox R. Friedrich O. (2019). Doing things with thoughts: brain-computer interfaces and disembodied agency. Philos. Technol. 32, 457–482. doi: 10.1007/s13347-018-0308-4

[ref139] Stipacek A. Grabner R. Neuper C. Fink A. Neubauer A. (2003). Sensitivity of human EEG alpha band desynchronization to different working memory components and increasing levels of memory load. Neurosci. Lett. 353, 193–196. doi: 10.1016/j.neulet.2003.09.044, PMID: 14665414

[ref140] Sweller J. (1988). Cognitive load during problem solving: effects on learning. Cogn. Sci. 12, 257–285. doi: 10.1016/0364-0213(88)90023-7

[ref141] Sweller J. (1994). Cognitive load theory, learning difficulty, and instructional design. Learn. Instr. 4, 295–312. doi: 10.1016/0959-4752(94)90003-5

[ref142] Sweller J. (2010). Element interactivity and intrinsic, extraneous, and germane cognitive load. Educ. Psychol. Rev. 22, 123–138. doi: 10.1007/s10648-010-9128-5

[ref143] Sweller J. (2020). Cognitive load theory and educational technology. Educ. Technol. Res. Dev. 68, 1–16. doi: 10.1007/s11423-019-09701-3

[ref144] Sweller J. van Merrienboer J. J. G. Paas F. G. W. C. (1998). Cognitive architecture and instructional design. Educ. Psychol. Rev. 10, 251–296. doi: 10.1023/A:1022193728205

[ref145] Sweller J. van Merriënboer J. J. G. Paas F. (2019). Cognitive architecture and instructional design: 20 years later. Educ. Psychol. Rev. 31, 261–292. doi: 10.1007/s10648-019-09465-5

[ref146] Tadson B. Boasen J. Courtemanche F. Beauchemin N. Karran A.-J. Léger P.-M.. (2023). “Neuro-adaptive Interface system to evaluate product recommendations in the context of E-commerce” in International conference on design science research in information systems and technology. eds. A. Gerber and R. Baskerville (Cham: Springer Nature Switzerland), 50–68.

[ref147] Tang J. Liu Y. Hu D. Zhou Z. (2018). Towards BCI-actuated smart wheelchair system. Bio Med. Eng. Online 17:111. doi: 10.1186/s12938-018-0545-x, PMID: 30126416 PMC6102906

[ref148] Tekin C. Braun J. Schaar M. V. D. (2015). eTutor: online learning for personalized education. 2015 IEEE international conference on acoustics, speech and signal processing (ICASSP), (pp. 5545–5549). IEEE.

[ref149] Tetzlaff L. Schmiedek F. Brod G. (2021). Developing personalized education: a dynamic framework. Educ. Psychol. Rev. 33, 863–882. doi: 10.1007/s10648-020-09570-w

[ref150] Tohidi H. Jabbari M. M. (2012). The effects of motivation in education. Procedia Soc. Behav. Sci. 31, 820–824. doi: 10.1016/j.sbspro.2011.12.148

[ref151] Torres-García A. A. Martínez-Santiago F. Montejo-Ráez A. Ureña-López L. A. (2023). Toward an educative EEG-based neuroIIR system for adapting contents. Int. J. Hum. Comput. Interact., 1–15. doi: 10.1080/10447318.2023.2275088

[ref152] Tuladhar A. M. Huurne N. Schoffelen J. M. Maris E. Oostenveld R. Jensen O. (2007). Parieto-occipital sources account for the increase in alpha activity with working memory load. Hum. Brain Mapp. 28, 785–792. doi: 10.1002/hbm.20306, PMID: 17266103 PMC6871495

[ref153] Urigüen J. A. Garcia-Zapirain B. (2015). EEG artifact removal—state-of-the-art and guidelines. J. Neural Eng. 12:031001. doi: 10.1088/1741-2560/12/3/031001, PMID: 25834104

[ref154] Van der Kleij F. M. Feskens R. C. Eggen T. J. (2015). Effects of feedback in a computer-based learning environment on students’ learning outcomes: a meta-analysis. Rev. Educ. Res. 85, 475–511. doi: 10.3102/0034654314564881

[ref155] van Merriënboer J. J. G. Ayres P. (2005). Research on cognitive load theory and its design implications for e-learning. Educ. Technol. Res. Dev. 53, 5–13. doi: 10.1007/BF02504793

[ref156] Vansteensel M. J. Klein E. van Thiel G. Gaytant M. Simmons Z. Wolpaw J. R.. (2023). Towards clinical application of implantable brain–computer interfaces for people with late-stage ALS: medical and ethical considerations. J. Neurol. 270, 1323–1336. doi: 10.1007/s00415-022-11464-6 36450968 PMC9971103

[ref157] Värbu K. Muhammad N. Muhammad Y. (2022). Past, present, and future of EEG-based BCI applications. Sensors 22:3331. doi: 10.3390/s22093331, PMID: 35591021 PMC9101004

[ref158] Venthur B. Blankertz B. Gugler M. F. Curio G. (2010). Novel applications of BCI technology: psychophysiological optimization of working conditions in industry. 2010 IEEE international conference on systems, man and cybernetics, (pp. 417–421). IEEE.

[ref159] Verkijika S. F. De Wet L. (2015). Using a brain-computer interface (BCI) in reducing math anxiety: evidence from South Africa. Comput. Educ. 81, 113–122. doi: 10.1016/j.compedu.2014.10.002

[ref160] Vijayalakshmi R. Nandagopal D. Dasari N. Cocks B. Dahal N. Thilaga M. (2015). Minimum connected component–a novel approach to detection of cognitive load induced changes in functional brain networks. Neurocomputing 170, 15–31. doi: 10.1016/j.neucom.2015.03.092

[ref161] Vincent J. L. Kahn I. Snyder A. Z. Raichle M. E. Buckner R. L. (2008). Evidence for a frontoparietal control system revealed by intrinsic functional connectivity. J. Neurophysiol. 100, 3328–3342. doi: 10.1152/jn.90355.2008, PMID: 18799601 PMC2604839

[ref162] Vlachogianni P. Tselios N. (2022). Perceived usability evaluation of educational technology using the system usability scale (SUS): a systematic review. J. Res. Technol. Educ. 54, 392–409. doi: 10.1080/15391523.2020.1867938

[ref163] Vygotsky L. S. Cole M. (1978). Mind in society: development of higher psychological processes. Cambridge, Massachusetts: Harvard University Press.

[ref164] Walter C. Rosenstiel W. Bogdan M. Gerjets P. Spüler M. (2017). Online EEG-based workload adaptation of an arithmetic learning environment. Front. Hum. Neurosci. 11:286. doi: 10.3389/fnhum.2017.00286, PMID: 28611615 PMC5448161

[ref165] Wang S. Gwizdka J. Chaovalitwongse W. A. (2016). Using wireless EEG signals to assess memory workload in the n-Back task. IEEE Transact. Hum. Mach. Syst. 46, 424–435. doi: 10.1109/THMS.2015.2476818

[ref166] Wang H. Lehman J. D. (2021). Using achievement goal-based personalized motivational feedback to enhance online learning. Educ. Technol. Res. Dev. 69, 553–581. doi: 10.1007/s11423-021-09940-3

[ref167] Wang Y. Nakanishi M. Zhang D. (2019). “EEG-based brain-computer interfaces” in Neural Interface: Frontiers and applications. ed. Zheng X. (Singapore: Springer), 41–65. 10.1007/978-981-13-2050-7_2 31729671

[ref168] Wickersham L. E. McGee P. (2008). Perceptions of satisfaction and deeper learning in an online course. Quart. Rev. Dist. Educ. 9:73.

[ref169] Wimmer G. E. Poldrack R. A. (2022). Reward learning and working memory: effects of massed versus spaced training and post-learning delay period. Mem. Cogn. 50, 312–324. doi: 10.3758/s13421-021-01233-7, PMID: 34519968 PMC8821056

[ref170] Wouters P. Paas F. van Merriënboer J. J. (2008). How to optimize learning from animated models: a review of guidelines based on cognitive load. Rev. Educ. Res. 78, 645–675. doi: 10.3102/0034654308320320

[ref171] Wu W. Wang B. Zheng W. Liu Y. Yin L. (2020). Higher education online courses personalized recommendation algorithm based on score and attributes. J. Phys. Conf. Ser. 1673:12025. doi: 10.1088/1742-6596/1673/1/012025

[ref172] Xia Q. Chiu T. K. Li X. (2023). A scoping review of BCIs for learning regulation in mainstream educational contexts. Behav. Inform. Technol. 43, 1–22. doi: 10.1080/0144929X.2023.2241559

[ref173] Xiao J. Wang M. Jiang B. Li J. (2018). A personalized recommendation system with combinational algorithm for online learning. J. Ambient. Intell. Humaniz. Comput. 9, 667–677. doi: 10.1007/s12652-017-0466-8

[ref174] Xu K. M. Koorn P. de Koning B. Skuballa I. T. Lin L. Henderikx M.. (2021). A growth mindset lowers perceived cognitive load and improves learning: integrating motivation to cognitive load. J. Educ. Psychol. 113, 1177–1191. doi: 10.1037/edu0000631

[ref175] Yuksel B. F. Oleson K. B. Harrison L. Peck E. M. Afergan D. Chang R.. (2016). Learn piano with BACh: An adaptive learning interface that adjusts task difficulty based on brain state. Proceedings of the 2016 CHI conference on human factors in computing systems, (pp. 5372–5384).

[ref176] Zammouri A. Moussa A. A. Mebrouk Y. (2018). Brain-computer interface for workload estimation: assessment of mental efforts in learning processes. Expert Syst. Appl. 112, 138–147. doi: 10.1016/j.eswa.2018.06.027

[ref177] Zander T. O. Kothe C. (2011). Towards passive brain–computer interfaces: applying brain–computer interface technology to human–machine systems in general. J. Neural Eng. 8:025005. doi: 10.1088/1741-2560/8/2/025005, PMID: 21436512

[ref178] Zander T. O. Kothe C. Welke S. Roetting M. (2009). “Utilizing secondary input from passive brain-computer interfaces for enhancing human-machine interaction” in Foundations of Augmented Cognition eds. D. D. Schmorrow, I. V. Estabrooke, and M. Grootjen (Berlin, Heidelberg: Neuroergonomics and Operational Neuroscience).

[ref179] Zhou Y. Xu T. Cai Y. Wu X. Dong B. (2017a). Monitoring cognitive workload in online videos learning through an EEG-based brain-computer Interface. Cham: Learning and Collaboration Technologies. Novel Learning Ecosystems.

[ref180] Zhou Y. Xu T. Cai Y. Wu X. Dong B. (2017b). Monitoring cognitive workload in online videos learning through an EEG-based brain-computer interface. Learning and collaboration technologies. Novel learning ecosystems: 4th international conference, LCT 2017, held as part of HCI international 2017, Vancouver, BC, Canada, July 9–14, 2017, proceedings, part I 4, (pp. 64–73). Springer International Publishing.

